# Identification of cyclins A1, E1 and vimentin as downstream targets of heme oxygenase-1 in vascular endothelial growth factor-mediated angiogenesis

**DOI:** 10.1038/srep29417

**Published:** 2016-07-08

**Authors:** Andrea Bauer, Hayley Mylroie, C. Clare Thornton, Damien Calay, Graeme M. Birdsey, Allan P. Kiprianos, Garrick K. Wilson, Miguel P. Soares, Xiaoke Yin, Manuel Mayr, Anna M. Randi, Justin C. Mason

**Affiliations:** 1 Vascular Sciences, Imperial Centre for Translational and Experimental Medicine, National Heart and Lung Institute, Imperial College London, Hammersmith Hospital, London, UK; 2 Instituto Gulbenkian de Ciência, 2780-156 Oeiras, Portugal; 3 King’s British Heart Foundation Centre, King’s College London, London, UK

## Abstract

Angiogenesis is an essential physiological process and an important factor in disease pathogenesis. However, its exploitation as a clinical target has achieved limited success and novel molecular targets are required. Although heme oxygenase-1 (HO-1) acts downstream of vascular endothelial growth factor (VEGF) to modulate angiogenesis, knowledge of the mechanisms involved remains limited. We set out identify novel HO-1 targets involved in angiogenesis. HO-1 depletion attenuated VEGF-induced human endothelial cell (EC) proliferation and tube formation. The latter response suggested a role for HO-1 in EC migration, and indeed HO-1 siRNA negatively affected directional migration of EC towards VEGF; a phenotype reversed by HO-1 over-expression. EC from *Hmox1*^−/−^ mice behaved similarly. Microarray analysis of HO-1-depleted and control EC exposed to VEGF identified cyclins A1 and E1 as HO-1 targets. Migrating HO-1-deficient EC showed increased p27, reduced cyclin A1 and attenuated cyclin-dependent kinase 2 activity. *In vivo*, cyclin A1 siRNA inhibited VEGF-driven angiogenesis, a response reversed by Ad-HO-1. Proteomics identified structural protein vimentin as an additional VEGF-HO-1 target. HO-1 depletion inhibited VEGF-induced calpain activity and vimentin cleavage, while vimentin silencing attenuated HO-1-driven proliferation. Thus, vimentin and cyclins A1 and E1 represent VEGF-activated HO-1-dependent targets important for VEGF-driven angiogenesis.

Although angiogenesis is fundamental to physiological processes in the adult, including wound healing and the menstrual cycle, it is also integral to the pathogenesis of atherosclerosis, diabetic retinopathy, inflammatory arthritis and malignant diseases. Investigation of angiogenesis as a potential therapeutic target has included strategies to either inhibit or enhance neovascularisation. However, clinically-derived benefit is relatively limited and different approaches and novel molecular targets are being sought^1^. Although the anti-oxidant, anti-apoptotic and anti-inflammatory enzyme heme oxygenase-1 (HO-1) has an established role in angiogenesis, understanding of associated downstream molecular mechanisms remains incomplete and the therapeutic potential of HO-1 minimally explored (reviewed in refs 2).

Extensive study has revealed a context-dependent effect of HO-1 activity in angiogenesis^2^,^4^. We initially proposed a dual role for HO-1 in chronic inflammation where HO-1 activity inhibits leukocyte migration, while promoting vascular endothelial growth factor (VEGF)-driven reparative angiogenesis^5^,^6^. Similar observations have been made in other settings^7^,^8^, and through the demonstration that HO-1 activity is protective against inflammatory arthritis^9^,^10^,^11^,^12^. HO-1 over-expression promotes endothelial cell (EC) proliferation and enhances development of endothelial tubules on Matrigel^13^. Subsequent pharmacological and molecular targeting experiments revealed the importance of HO-1 activity in growth-factor driven EC cell cycle progression, proliferation and capillary formation^5^,^14^,^15^. *Hmox-1*-deficient mice exhibit reduced endothelial proliferative responses to growth factors, attenuated tube formation and impaired wound healing^16^,^17^. In addition, *Hmox-1*^−/−^ EC are more susceptible to pro-apoptotic stimuli than *Hmox-1*^+/+^ EC^18^. Thus, increased resistance to apoptosis may add to the pro-angiogenic influence of HO-1.

A complex relationship exists between VEGF and HO-1. First, VEGF synthesis can be altered by HO-1 activity. HO-1 inhibition impairs VEGF release in response to prostaglandin J_2_ (PGJ_2_), hemin and hypoxia, while increased HO-1 activity or delivery of carbon monoxide (CO) enhances VEGF release^19^,^20^,^21^. Second, VEGF is able to increase HO-1 expression and activity^5^,^22^, suggesting the presence of a positive feedback pathway and endogenous amplification^6^,^23^. These data have been reinforced and extended by two recent studies. In the first, treatment of EC with VEGF or stromal cell-derived factor-1α (SDF-1α) led to translocation of nuclear factor E2-related factor 2 (Nrf2) and induction of HO-1. Subsequent experiments confirmed the importance of Nrf2 activation for angiogenesis^24^. Meanwhile, the hypoxic induction of miR-101 regulates angiogenesis by binding to cullin 3 and stabilising Nrf2, leading to activation of an HO-1/VEGF/endothelial nitric oxide synthase (eNOS) pathway^25^.

The next challenge is to identify the target genes acting downstream of HO-1 and its products, and ultimately to determine whether these pathways might be targeted therapeutically. Analysis of HO-1 enzymatic product CO has shed some light and suggests CO may contribute to the proliferative and pro-migratory actions of HO-1^14^,^20^,^26^. CO rapidly activates Ras homolog gene family member A (RhoA) leading to Akt and eNOS phosphorylation and increased nitric oxide biosynthesis^26^. The importance of RhoA implies a role for cytoskeletal and cell cycle regulators. Indeed, the pro-angiogenic actions of SDF-1α are mediated at least in part through activation of HO-1 and CO release, with subsequent phosphorylation of the cytoskeletal regulator vasodilator-stimulated phosphoprotein (VASP)^16^. Delivery of CO reversed the angiogenic defects seen in SDF-1α-treated *Hmox1*^−/−^EC and aortic rings^16^. However, context-dependent, cell-type specific actions have also been reported, with HO-1 and CO able to either enhance or inhibit angiogenesis under specific circumstances^14^,^20^,^26^,^27^, particularly in the tumour microvasculature^28^. These findings, which likely reflect differences in the local microenvironment, including the presence or absence of individual growth factors and pro-inflammatory responses, serve to further emphasise the dual role of HO-1 in different settings^6^, and the need for improved understanding of molecular mechanisms.

In the current study, a gain and loss of function approach employed targeted genomic and proteomic techniques to identify HO-1 downstream targets that regulate angiogenesis. The data generated demonstrate that cyclins A1 and E1, and the structural protein vimentin, are HO-1-dependent proteins that play an important role in VEGF-driven angiogenic responses including cell proliferation and migration.

## Materials and Methods

### Reagents

Antibodies against Bcl-2, cyclin A1, proliferating cell nuclear antigen (PCNA) and retinoblastoma (Rb) protein were purchased from Santa Cruz Biotechnology (California, USA). Anti-HO-1 and anti-HO-2 were from Stressgen (Exeter, UK). Anti-phospho Rb^Thr821^ and anti-vimentin were from Invitrogen (Paisley, UK) and Abcam (Cambridge, UK) respectively. Anti-α-tubulin and hemin were from (Sigma-Aldrich (Poole, UK) and anti-GAPDH from Millipore (Feltham, UK). HO inhibitor zinc (II) protoporphyrin IX chloride (ZnPPIX) was from Frontier Scientific (Logan, UT).

### Mice

All animals were studied according to the guidelines from Directive 2010/63/EU of the European Parliament. *Hmox1*^−/−^ mice (back-crossed 10 generations into the C57BL/6 background) were originally generated by Dr S-F Yet (Pulmonary and Critical Care Division, Brigham and Women’s Hospital, Boston, MA). The colony was maintained by *Hmox1*^+/−^ mating, under specific pathogen-free conditions following guidelines from the Animal User and Institutional Ethical Committees of the Instituto Gulbenkian de Ciência. Female C57BL/6 mice were purchased from Harlan Laboratories (Oxford, UK) and housed under controlled conditions in microisolator cages with autoclaved bedding, and ethical approval from Imperial College London under UK Home Office Licence number PPL70/6722. Irradiated food and drinking water were available *ad libitum*.

### Matrigel plug assay

Angiogenesis was quantified as previously described^5^,^29^. Female C57BL/6 mice (10 weeks old) were anaesthetised with isoflurane and injected subcutaneously with Matrigel (BD Biosciences, San Jose, CA), along the abdominal midline. Matrigel (8.13 mg/ml) was mixed with heparin (64 U/ml), VEGF (40 ng/mL) or vehicle. In gene silencing studies 2 μM murine siRNA (siHO-1 pool, siCyclin A1 pool, or control siRNA) was incorporated in the Matrigel and PBS added to a final volume of 0.5 ml. In gene over-expression studies, Ad-HO-1 or Ad0 (1 × 10^9^ ifu/ml) were combined with Matrigel preparations as described above. Plugs were harvested after 7 days from CO_2_-euthanised mice, fixed in 4% (w/v) paraformaldehyde, transferred to 70% ethanol, embedded in paraffin and processed for hematoxylin and eosin staining. Neovessels were identified by the presence of nucleated cells in the vascular wall surrounding a lumen containing erythrocytes. The area of the Matrigel plug occupied by neovessels was quantified using ImageJ 1.29 software (National Institutes of Health (NIH), Bethesda, MD) and expressed as a percentage of the total area in 5 separate fields of view using a 20x objective lens.

### Human and murine cell culture

The use of human EC was approved by Hammersmith Hospitals Research Ethics Committee (ref. 06/Q0406/21). Human umbilical vein EC (HUVEC) and human dermal microvascular endothelial cell line HMEC-1 (a gift from Dr E. Ades, CDC Atlanta), were cultured as described^30^. Murine EC (MEC) were isolated from *Hmox1*^−/−^ and *Hmox1*^+/+^ littermate controls as previously reported^31^,^32^.

### Immunoblotting

Immunoblotting of EC lysates was performed as described previously^33^. To control for sample loading, membranes were re-probed with an α-tubulin or GAPDH Ab. Relative levels of protein expression were quantified using ImageJ.

### siRNA transfection

siRNA was introduced into EC using GeneFECTOR (3:50) (VennNova, Parkland, Fl). GeneFECTOR and siRNA (40 nM final) were diluted separately in Opti-MEM I (ThermoFisher Scientific, Runcorn, UK. Transfection solutions were added to HUVEC cultured in Opti-MEM I medium. After incubation for 6 h, culture medium was replaced with EBM2 medium (Lonza, Wokingham, UK) overnight and then with M199/10% FBS. Single sequence siRNA against human HO-1 (siHO-1 seq2) was purchased from Qiagen (Manchester, UK). The siRNA pools of 4 sequences against human HO-1, vimentin and cyclin A1 and murine HO-1 and cyclin A1 were from Dharmacon (Lafayette, CO). A non-targeting siRNA *Silencer* Negative Control #1 was purchased from Ambion (Austin, Texas). 3′-end AlexaFluor 488-labelled siRNA against HO-1 and the AllStars Negative Control siRNA AlexaFluor 488 were from Qiagen.

### Adenoviral infection

The recombinant adenovirus expressing *Hmox1* (Ad-HO-1) and the Ad0 control adenovirus were amplified in human embryonic kidney 293A cells, purified and titrated as described^32^. HUVEC were infected by incubation with adenovirus in serum-free M199 for 2 h at 37 °C and maintained in M199 containing 10% fetal FBS and 7.5 μg/ml endothelial cell growth factor (Sigma-Aldrich). Optimal multiplicity of infection (MOI) for Ad-HO-1 was previously determined by immunoblotting^32^.

### Proliferation, apoptosis and cell cycle assays

HUVEC proliferation was quantified using the Cell Proliferation BrdU ELISA (Roche Diagnostics, Burgess Hill, UK) according to the manufacturers’ instructions. EC were labelled with 10 μM BrdU for 3 hours, fixed and denatured with FixDenat solution, and incubated with an anti-BrdU-peroxidase mAb for 90 min. Immune complexes were detected by tetramethyl-benzidine and quantified using a plate reader measuring absorbance at 370 nm and a reference wavelength at 494 nm. In addition, proliferation was assessed by flow-cytometry using an anti-PCNA antibody. For cell cycle analysis HUVEC transfected with HO-1 specific or control siRNA were treated with 25 ng/mL VEGF or vehicle for 16 h followed by fixation in 70% ethanol and staining with a propidium iodide (PI) solution composed of 50 μg/ml propidium iodide, 20 U/ml RNase (both Sigma-Aldrich), 18 mg/ml EDTA, and 0.1% Triton X-100 for 45 min at 37 °C. Apoptotic cells were quantified by measuring the sub-G_1_ population by flow-cytometry.

### Migration and tube formation assays

HUVEC monolayers were scratched and the migration path of the leading edge assessed by live cell imaging (LSM510 Meta; Zeiss, Oberkochen, Germany) and further analysis using ImageJ 1.29 software and the Ibidi Chemotaxis and Migration tool (Ibidi GmbH, München, Germany). Chemotatic behaviour of control or HO-1 siRNA-transfected EC was assessed using the μ-slide chemotaxis assay (Ibidi). HUVEC were cultured on collagen IV-coated μ-slides in EGM-2 supplemented with 10% FBS and allowed to adhere for 3 hours. One reservoir was filled with growth factor-free EBM/2% FBS and the second reservoir with VEGF-supplemented medium (25 ng/ml). Directional migration was assessed after 16 hours and tracked with the aid of time-lapse microscopy. Formation of tubular structures was studied on growth factor-reduced Matrigel. EC transfected with control or HO-1-specific siRNA were seeded onto Matrigel in growth factor-free EBM-2/2% FBS. Tube formation was assessed by phase-contrast microscopy after 16 hours and quantified using the ImageJ plugin NeuronJ (NIH).

### Immunofluorescence

HUVEC cultured on glass coverslips were fixed in methanol for 15 min at −20 °C, permeabilized with 0.5% Triton-X 100, and stained with anti-vimentin mAb overnight at 4 °C and Alexa Fluor 568-labelled goat anti-mouse IgG_1_ for 1 hour. Nuclei were visualized with Draq 5 (Cell Signaling Technology, Danvers, MA). Coverslips were mounted onto glass slides and Z-stack projections captured by confocal microscopy.

### *In vitro* adhesion assay

Control or HO-1 specific siRNA-treated HUVEC were labelled with 5-chloromethylfluoresein diacetate (6.25 μM) (Invitrogen Cell Tracker Green, Invitrogen, Paisley, UK) for 30 min and re-seeded at 20,000 cells per well of 96-well plates coated with gelatin or collagen type I (BD Biosciences) and cultured in EBM-2 media supplemented with VEGF (25 ng/ml) or vehicle for 40 min. Total cellular fluorescence was recorded using absorption and emission settings at 492 and 517 nm respectively, and bound EC quantified as a percentage of the total cells seeded.

### Senescence-associated beta-galactosidase (SA-β-gal) staining

HUVEC (passage 2–4) were fixed with 0.5% glutaraldehyde/PBS and incubated with 20 mg/ml 5-bromo-4-chloro-3-indolyl β-D-galactosidase (X-gal) in N,N-dimethylformamide, 100 mM potassium hexacyano-ferrate (III) (K_3_[Fe(CN)_6_]) and 100 mM potassium hexacyano-ferrate (II) trihydrate (K_3_[Fe(CN)_6_])*3H_2_O (all Sigma-Aldrich) in PBS for 24 hours at 37 °C. HUVEC cultured for >12 passages acted as a positive control, with senescent cells identified by phase-contrast microscopy.

### Calpain activity

Control or HO-1 specific siRNA-transfected HUVEC were loaded with 30 μM 7-amino-4chloromethycoumarin (tBoc-LM-CMAC; Invitrogen) in serum-free medium for 30 min prior to VEGF treatment. Fluorescence was measured on a CyAN ADP analyzer (Beckman Coulter, High Wycombe, UK) using the Violet-1 filter (FL6).

### Analysis of PCR Arrays

SABiosciences Human Cytoskeleton Regulators RT^2^ Profiler PCR Array (Qiagen) was used as per manufacturers’ instructions. HUVEC from 3 separate donors were transfected with HO-1 or control siRNA and exposed to VEGF for 16 hours before lysis in RLT Buffer. Lysate RNA was isolated using the RNeasy Mini Kit (Qiagen) and quantified by Nanodrop analysis. Ribosomal band integrity was determined on an Agilent 2100 BioAnalyzer, using an RNA 6000 Nano LabChip (Agilent Technologies, Santa Clara, CA.).

### Difference in-gel electrophoresis (DIGE) and mass spectrometry

HUVEC transfected with HO-1 or control siRNA and exposed to VEGF for 16 hours were lysed with DIGE lysis buffer containing 8 M urea, 4% CHAPS, protease inhibitors and 30 mM Tris (pH = 8.5). Cell lysates were subjected to difference in-gel electrophoresis (DIGE) followed by protein identification using nanoflow liquid chromatography (LC) and tandem mass spectrometry. The samples were labelled with Cy3 or Cy5 (GE Healthcare, Amersham, UK) and 40 ug of proteins were mixed together with a Cy2-labelled pooled standard. Protein samples were mixed with 2x DIGE buffer (8 M urea, 2% DTT, 4% CHAPS, 2% pharmalytes 3–10 for IEF) and further diluted with rehydration buffer (8 M urea, 0.5% CHAPS, 0.2% DTT, 0.2% pharmalytes, and trace bromophenol blue) to 450 ul. The samples were applied to IPG strips (18 cm, 3–10 NL, GE Healthcare) and rehydrated overnight. Strips were focused at 0.05 mA/IPG strip for 28 kVh at 20 °C overnight and equilibrated in buffer (6 M urea, 2% SDS, 30% glycerol, 50 mM Tris, pH = 8.8, trace bromophenol blue) with 10 mg/mL DTT for 15 min and with 48 mg/mL iodoacetamide for 15 min. The IPG strips were transferred to large format polyacrylamide gels (12% total acrylamide, 2.6% cross-linking) and underwent SDS-PAGE. Fluorescent images were acquired using an Ettan DIGE imager (GE Healthcare) and gels were silver-stained to locate differentially expressed protein spots. Fluorescent images were analysed by DeCyder software (version 7.0, GE Healthcare). Protein spots with fold change >1.2 or <−1.2 and p < 0.05 were excised manually. Selected spots were subjected to overnight in-gel tryptic digestion using a robot (ProGest, Digilab, Artisan Technology group, Champaign, IL). Tryptic peptides were separated by nanoflow LC (Ultimate 3000 RSLCnano, ThermoFisher) on a reverse-phase C18 column (PepMap100, C18, 25 cm × 75 um) interfaced to a LTQ Orbitrap XL mass spectrometer (ThermoFisher) with full scan mass-to-charge (m/z) range of 400–1600 followed by 6 data-dependent MS2 scans with dynamic exclusion enabled. A database search was performed using Mascot server (Matrix Science Ltd, London, UK) against a human database (UniProt/SwissProt 57.15, 20266 protein entries) with the following parameters: precursor mass tolerance 10 ppm, fragment mass tolerance 0.8 Da, carbamidomethylation on cysteine as fixed modification, oxidation on methionine as variable modification, maximum 2 miss cleavages were allowed. Scaffold software (Proteome Software, Portland, OR) was used to validate MS/MS-based peptide and protein identity with the following filters: peptide probability >95%, protein probability >99%, minimum number of peptides per protein > = 2^34^. Differentially expressed proteins were analyzed using Database for Annotation, Visualization and Integrated Discovery (DAVID) Bioinformatics Resources 6.7 (National Institute of Allergy and Infectious Diseases, NIH) and grouped according to gene ontology (GO).

### Statistics

Numerical data are presented as the mean of individual experiments ± standard error (SEM). Data were grouped according to treatment and analysed using GraphPad Prism Software (San Diego, CA, USA). Differences between treatments were evaluated using either an unpaired Student’s *t*-test, or a one-sample *t*-test for comparison of two samples. For evaluation of three or more samples the one-way analysis of variance (ANOVA) was used. A Bonferroni correction was used to correct for multiple comparisons. Differences were considered significant at a *P* < 0.05.

## Results

### Role of HO-1 in VEGF-induced EC proliferation and apoptosis

To investigate the role of HO-1 in VEGF-mediated endothelial proliferation and resistance to apoptosis, a gain and loss of function approach using specific siRNA-mediated depletion and adenoviral over-expression was adopted. To validate HO-1 siRNAs, hemin treatment of HUVECs was used to induce HO-1 protein. A single HO-1 siRNA oligonucleotide (Seq 2) and a pool of 4 targeted siRNAs reduced HO-1 by 90%, while having no effect on HO-2 (Supplementary Figure 1A,B and data not shown). Infection of EC with Ad-HO-1 increased HO-1 protein after 24 h, maximal at an MOI of 100 ifu/cell (Supplementary Figure 1C).

**Figure 1:**
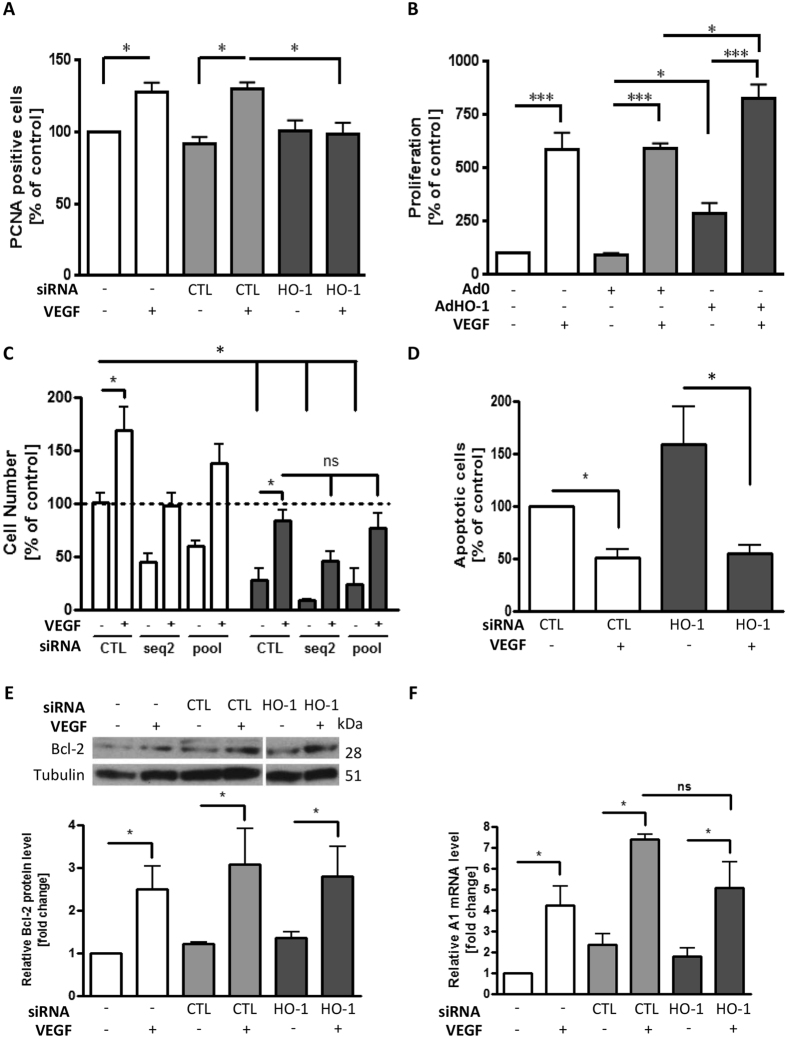
siRNA-mediated HO-1 depletion abrogates VEGF-induced HUVEC proliferation. (**A**) HUVEC were transfected with control siRNA (CTL) or HO-1-specific siRNA prior to culture in the presence or absence of VEGF (25 ng/ml) for 48 h. Proliferation was quantified by flow-cytometric analysis of PCNA staining. (**B**) HUVEC were left uninfected or infected with the Ad0 control adenovirus or AdHO-1 (multiplicity of infection (MOI) 100 ifu/cell). EC were then cultured in the absence or presence of VEGF for 48 h and proliferation quantified by BrdU ELISA. (**C**) HUVEC were treated with vehicle alone (dotted line) or transfected with control siRNA (CTL) or HO-1 specific siRNA (seq2 or 4 pooled sequences (pool)). Cells were cultured in M199 medium/10% FCS (white bars) or serum-starved in M199/0.1% BSA in the absence or presence of VEGF (25 ng/ml) for 48 h (dark bars). Cell number was assessed by MTS-assay. (**D**) HUVEC were transfected with control (CTL) or pooled HO-1 siRNA oligos (HO-1) as above prior to culture with or without VEGF and serum starvation. Sub-G1 apoptotic EC were identified by propidium iodide staining and quantified by flow-cytometry, with data expressed as a percentage of the apoptosis seen in control siRNA-treated serum-starved cells. (**E,F**) HUVEC were transfected with control or pooled HO-1 siRNA (HO-1) and cultured in the absence or presence of VEGF for 48 h, with (E) Bcl-2 protein analysed by immunoblotting and quantified by densitometry, and (**F**) A1 mRNA quantified by qRT-PCR. Data are presented as mean ± SEM (n ≥ 4 experiments), *p < 0.05, **p < 0.01, ***p < 0.001, ns = not significant.

Endothelial proliferation was quantified by BrdU incorporation and flow-cytometric analysis of PCNA. The presence of HO antagonist ZnPPIX exerted a concentration-dependent inhibitory effect on VEGF-induced HUVEC proliferation. A 7-fold increase in proliferation was reduced to only 1.5–2-fold by ZnPPIX (20 μM) (Supplementary Figure 1D). VEGF treatment for 48 h increased EC PCNA expression by up to 30%, a response completely attenuated by HO-1 depletion (p < 0.05) (Fig. 1A). Adenoviral over-expression of HO-1 also increased EC proliferation by up to 3-fold and further increased the VEGF-induced proliferative response from 6- to 8-fold, when compared with the control adenovirus (Fig. 1B).

Next, an MTT cell survival assay was used to investigate the role of HO-1 in VEGF-mediated cytoprotection. First, both HO inhibitor ZnPPIX and HO-1 silencing reduced the basal number of EC by up to 50%. VEGF increased the EC number by 50–70% at 24 h and this response was significantly attenuated by ZnPPIX and HO-1 siRNA (p < 0.05) (Supplementary Figure 1E and Fig. 1C). Serum starvation (grey bars) reduced EC survival by 75%, a response almost completely reversed by the presence of VEGF. However, the changes in VEGF-mediated cytoprotection seen following the inclusion of ZnPPIX or the depletion of HO-1 failed to reach significance, so distinguishing the protective response from EC proliferation (Supplementary Figure 1E and Fig. 1C). These data were confirmed using propidium iodide labelling followed by flow-cytometric analysis of the sub-G1 apoptotic cell population. When compared to serum-starved control siRNA-transfected EC, VEGF reduced apoptosis by 50%, while HO-1 depletion increased apoptosis by 1.5-fold. However, as before, VEGF-mediated cytoprotection was not attenuated by HO-1 silencing (Fig. 1D). In support of these findings, analysis of VEGF-inducible anti-apoptotic genes showed that neither the 7-fold induction of A1, nor the 3-fold induction of Bcl-2 in VEGF-treated HUVEC transfected with control siRNA was impaired by HO-1 silencing (Fig. 1E,F).

### HO-1 influences directional migration of EC

**Figure 2:**
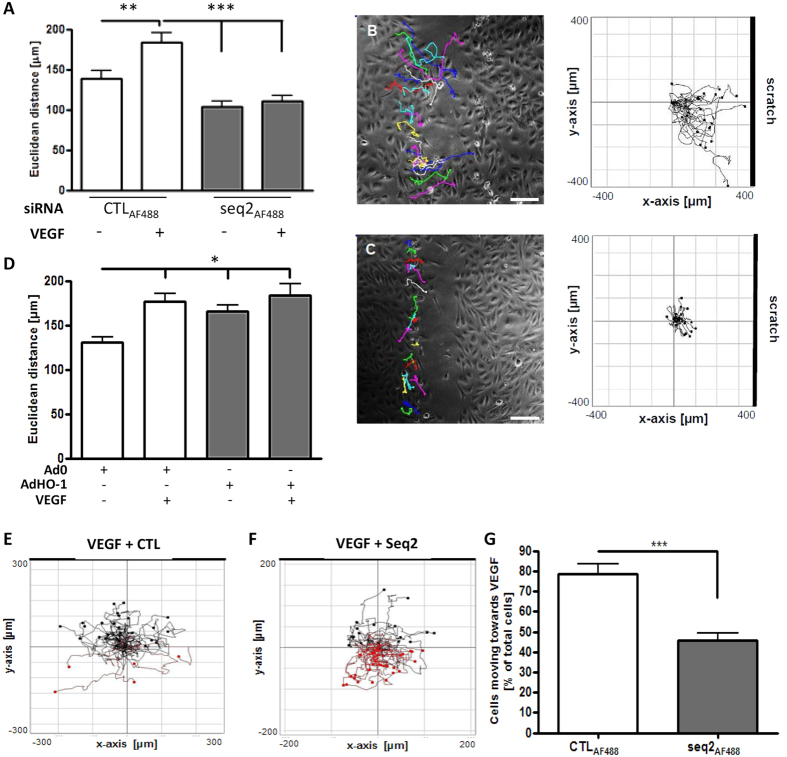
HO-1 inhibition impairs EC migration *in vitro*. HUVEC were transfected with 3′-AlexaFluor488-labelled control siRNA (CTLAF_488_) or HO-1-specific siRNA (seq2AF_488_) and (**A**) Confluent EC monolayers were scratched and migration (Euclidean distance) was assessed by live cell imaging in the absence or presence of VEGF (25 ng/ml) for 16 h. (**B,C**) Illustrate directional migration, with images representative of n = 3 experiments, (**B**) Ctl siRNA and (**C**) HO-1 siRNA, with histograms plotting migration of individually tracked cells. Bar = 50 μm. (**D**) HUVEC were infected with Ad0 or AdHO-1 (MOI 100) and cultured for 16 h. Confluent cell monolayers were scratched and migration assessed in the absence or presence of VEGF as above for 16 h. (**E,F**) HUVEC were transfected with CTLAF_488_ (**E**), or (**F**) HO-1 siRNA (seq2AF_488_) and re-seeded onto collagen IV-coated Ibidi chemotaxis μ-slides. The upper reservoir contained EBM-2 medium with 25 ng/ml VEGF and the lower reservoir plain EBM-2. Tracking of fluorescent transfected cells was captured by time-lapse microscopy every 15 mins for 16 h. Migration paths along the VEGF gradient are shown in black and away in red. Plots are representative of 3 experiments and (**G**) shows quantitative data presented as mean ± SEM (n = 3). Migration was quantified using ImageJ software (Manual Tracking, Chemotaxis and Migration tool). Data are presented as mean ± SD (n ≥ 3 experiments), *p < 0.05, **p < 0.01, ***p < 0.001.

Directional migration of EC is an essential component of angiogenesis. A scratch assay was employed to determine the role of HO-1 in this process. To identify and track individual HUVEC, AlexaFluor488-labelled HO-1 siRNA (seq2_AF488_) or control siRNA (CTL_AF488_) was used (Supplementary Figure 1B). VEGF increased the linear distance between the starting and end points of the migration pathway (Euclidean distance) from 139 ± 9.8 to 183 ± 12.5 μm (p < 0.01). VEGF-enhanced HUVEC migration was reduced by HO-1 depletion to 111 ± 7.4 μm (p < 0.001) (Fig. 2A), with comparable results seen with HMEC (Supplementary Figure 2A). Plotting the individual paths tracked by the transfected cells showed that VEGF treatment increased migration in the direction of the scratch (Fig. 2B). HO-1 depletion inhibited this effect, with cell migration markedly restricted (Fig. 2C). Although over-expression of HO-1 enhanced cell migration to a similar extent to VEGF (177 ± 8.9 μm), in combination no synergistic or additive effect was observed (184 ± 12.6 μm) (Fig. 2D).

The Ibidi μ-slide chemotaxis assay was used to assess the role of HO-1 in mediating chemotactic migration of EC. HUVEC were transfected with seq2_AF488_ or control CTL_AF488_ and migration towards VEGF or control media containing reservoirs was captured by time-lapse microscopy. Single cell tracking of fluorescent cells towards the stimulus is shown in black, and away from the stimulus in red (Fig. 2E,F). The significant majority of control-transfected cells migrated towards VEGF, whereas HO-1 depletion impaired chemotaxis, decreasing migration towards VEGF by 40% (p < 0.001) (Fig. 2G).

### HO-1 inhibition impairs EC tubule formation

**Figure 3:**
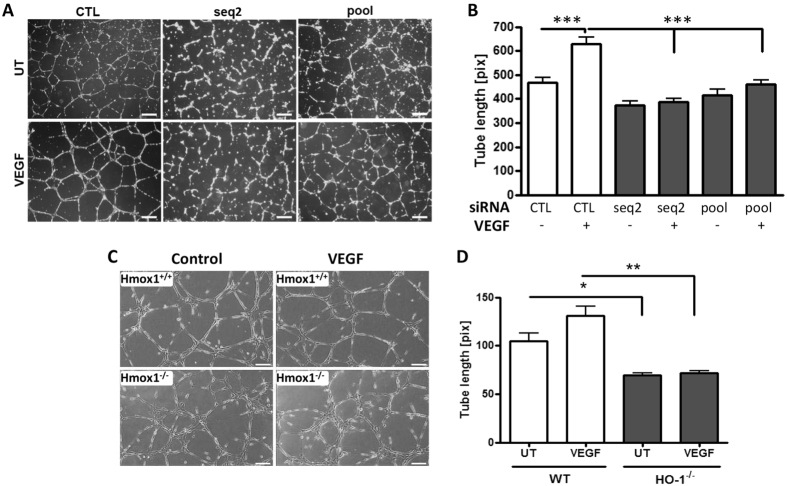
HO-1 inhibition impairs directional migration of EC. (**A**) HUVEC were transfected with control siRNA (CTL), HO-1 siRNA (seq2) or pooled HO-1 siRNAs (pool). Cells were seeded onto growth factor-reduced Matrigel in the presence or absence of VEGF for 16 h. Images from five fields per view were captured, and (**B**) tube length was quantified using ImageJ software (NeuronJ). (**C**) Murine EC from *Hmox1*^−/−^ mice or *Hmox1*^+/+^ littermate controls were seeded onto growth factor-reduced Matrigel in the presence or absence (UT) of VEGF for 16 h. Images from five fields per view were captured and (**D**) tube length quantified using ImageJ. Pictures are representative of 3 experiments (Bars = 50 μm). Data are presented as mean ± SEM (n = 3 experiments), *p < 0.05, **p < 0.01, ***p < 0.001.

Formation of tubules on Matrigel was used as a functional end-point for changes in EC migration. Exposure of control siRNA-transfected HUVEC to VEGF accelerated tube formation and increased average tube length by 34% from 469 ± 19.3 to 629 ± 26.7 pixels. HO-1 depletion reversed this VEGF response in HUVEC, reducing tube length to a minimum of 387 ± 14.2 (Fig. 3A,B), with the same effect observed with HMEC (Supplementary Figure 2B). Although murine EC isolated from *Hmox1*^−/−^ mice and wild-type littermates formed tubular networks on Matrigel, the former were less well differentiated. Furthermore, WT EC responded to VEGF with increased tube length of 25%, while *Hmox1*^−/−^ tubules remained shorter 69 ± 2.3 vs 105 ± 7.9 μm and less well formed, with clusters of EC observed. They also failed to respond to VEGF (72 ± 1.9 μm) (Fig. 3C,D).

### HO-1 depletion predisposes to cell cycle arrest

In light of the EC migration data obtained, a PCR array incorporating 84 genes involved in cytoskeletal regulation was initially chosen to investigate downstream effectors of HO-1 in VEGF-driven responses. Pooled HUVEC, derived from 3 donors, were transfected with control or HO-1 siRNA and treated with VEGF for 16 h prior to analysis. The expression of four transcripts was increased >2.0-fold in HO-1-depleted EC: Actin related protein 2/3 complex, subunit 2, Citron (rho-interacting, serine/threonine kinase 21), Fascin homolog 2, actin-bundling protein and Protein phosphatase 1, regulatory (inhibitor) subunit 12B (Supplementary Table 1). A single gene, the positive cell cycle regulator cyclin A1, was found to be significantly down-regulated by HO-1 depletion (fold change 0.44). As this latter response suggested cyclin A1 is positively regulated by HO-1, it was selected for further analysis.

**Figure 4:**
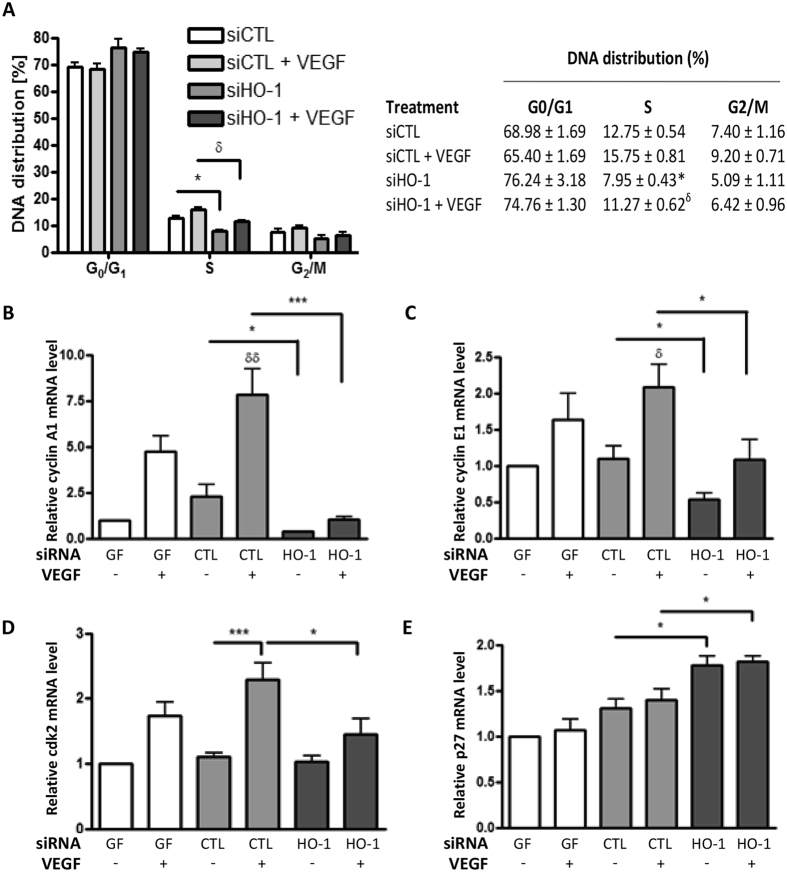
HO-1-deficiency results in endothelial cell cycle arrest. (**A**) Control siRNA (siCTL)-transfected or pooled siHO-1-transfected HUVEC were left untreated or treated with 25 ng/ml VEGF for 48 h prior to propidium iodide staining and flow-cytometric analysis of DNA distribution. Data is presented as mean ± SEM (n = 3 experiments), *p < 0.05 vs siCTL, ^δ^*p* < 0.05 vs siCTL + VEGF. (**B**) HUVEC were exposed to geneFECTOR alone (GF) or transfected with control siRNA (CTL) or pooled HO-1 siRNA (HO-1). Cells were cultured in the presence or absence of VEGF for 24 h prior to mRNA quantification by qRT-PCR of (**B**) cyclin A1, (**C**) cyclin E1, (**D**) cyclin-dependent kinase 2 (cdk2) or (**E**) p27. Data are presented as mean ± SEM (n = 3 experiments), *p < 0.05, **p < 0.01. ^δδ^*p* < 0.01 vs untreated siCTL-transfected cells.

First, the effect of HO-1 depletion on cell cycle progression was analysed. In response to VEGF, control siRNA-transfected cells progressed from G_0_/G_1_ to S-phase as indicated by an increase in DNA content from 12.75 ± 0.54% to 15.75 ± 0.81%. This response was significantly diminished by HO-1 depletion, both in the absence and presence of VEGF, resulting in S-phase DNA distributions of 7.95 ± 0.43% and 11.27 ± 0.62% respectively (Fig. 4A). To investigate a potential role for premature cellular senescence, EC were stained for SA-β-gal activity. Prolonged culture of HUVEC for 12 passages resulted in an 80% increase in SA-β-gal positive cells in the absence and presence of VEGF, when compared to cells cultured for 4 passages. However, HO-1 silencing had no significant effect on EC senescence (Supplementary Figure 3), suggesting that cell cycle arrest in HO-1-depleted cells is not the result of premature senescence. Next, the induction of cyclin A1 by VEGF and the inhibition of this response by HO-1 silencing was further validated (Fig. 4B). Additional study of cyclins not included in the PCR array showed that cyclin E1 responded similarly to A1 (Fig. 4C), while cyclin B1 and D1 were induced by VEGF but not affected by HO-1 depletion (Supplementary Figure 3B,C).

### Relationship between cyclin A1, Cdk2 and HO-1

Cyclins A1 and E1 form complexes with and regulate the activity of cyclin-dependent kinase2 (Cdk2). Cdk2 was increased in VEGF-treated HUVEC, a response attenuated by specific depletion of HO-1 (Fig. 4D). This may in turn reflect inhibition by p27, as analysis of the innate Cdk2 inhibitors p21 and p27 showed that, while VEGF treatment had no significant effect on p21 or p27 expression *per se*, HO-1 depletion led to a modest but significant increase in p27 (Fig. 4E), a response not seen for p21 (not shown).

**Figure 5:**
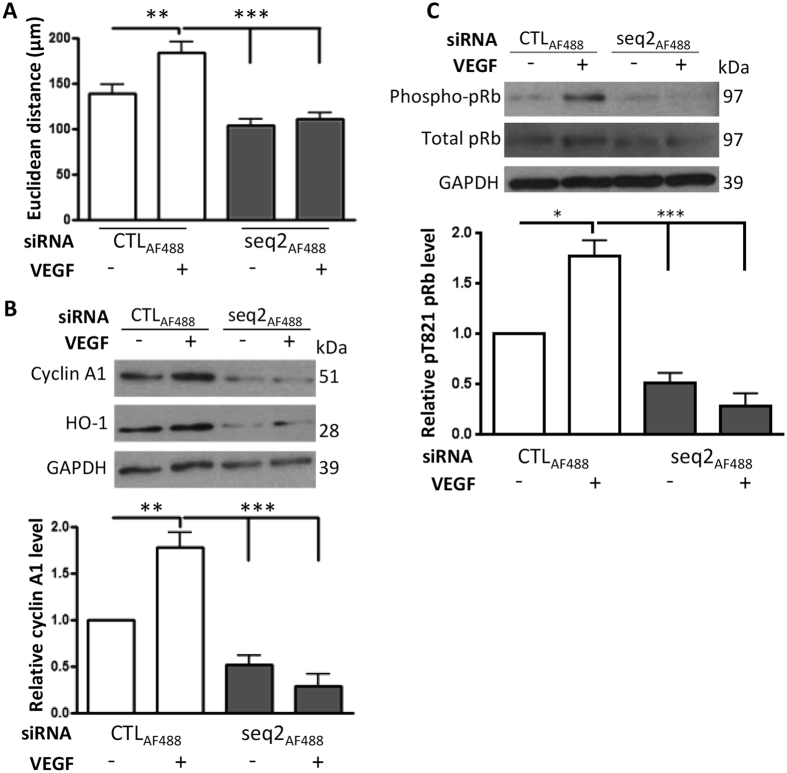
Cyclin A1 is reduced in migrating HO-1-deficient EC. HUVEC were transfected with CTLAF_488_ or HO-1 siRNA (seq2AF_488_). Monolayers were scratched and treated with vehicle alone or VEGF (25 ng/ml) for 16 h (**A**) Migration was quantified by live cell imaging using ImageJ. EC were then harvested, lysed and immunoblotted for: (**B**) cyclin A1 and (**C**) retinoblastoma protein (pRb) and phospho-pRb^pT821^. The histograms show corresponding densitometry data corrected for the GAPDH loading control and with phospho-pRb expressed relative to total pRb. Data are presented as mean ± SEM (n = 3 experiments), *p < 0.05, **p < 0.01; ***p < 0.001.

**Figure 6:**
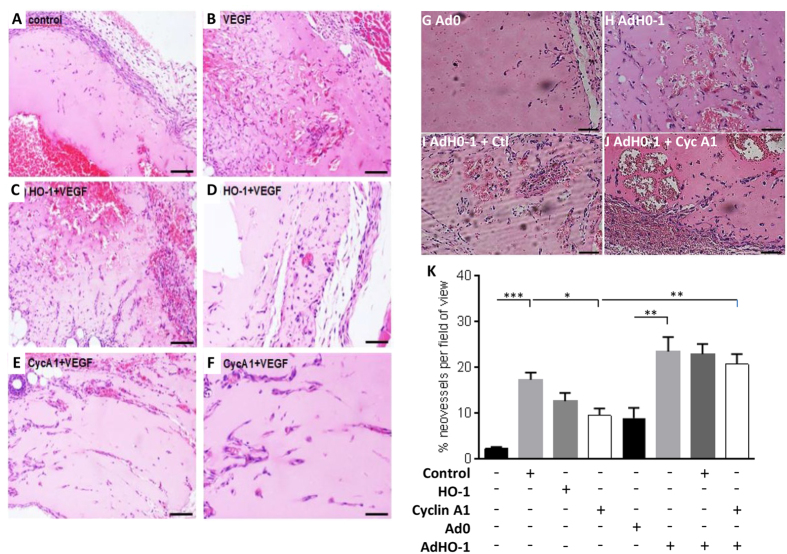
Silencing of cyclin A1 impairs angiogenesis *in vivo*. Female C57BL/6 mice (10 weeks old, n = 3 per treatment) were injected with Matrigel preparations containing: (**A**) vehicle control, (**B**) VEGF (40 ng/ml) with 2 μM control siRNA (CTL), (**C,D**) VEGF with murine HO-1 siRNA (HO-1), (**E,F**) VEGF with murine CycA1 siRNA (CycA1). (**A,B,C,E**) 10x magnification, bar = 50 μm (**D,F**) 20x magnification, bars = 100 μm. In (**G–J**) Matrigel plugs contained (**G**) Ad0 control, (**H**) AdHO-1, (**I**) AdHO-1 + Ctl siRNA, (**J**) AdHO-1 + CycA1 siRNA. Representative images (10x magnification, bars = 50 μm). (**K**) Area occupied by neovessels quantified using ImageJ software. Data are presented as mean ± SEM (n = 3 experiments). *p < 0.05, **p < 0.01; ***p < 0.001.

Migrating EC were studied to investigate the functional relationship between HO-1, cyclin A1 and Cdk2 activity. As above, HO-1 depletion inhibited the VEGF-induced increase in the Euclidean distance (Fig. 5A) and the induction of cyclin A1 (Fig. 5B). Moreover, cyclin A1 levels were significantly reduced in the HO-1 depleted EC by 50–75% (Fig. 5B). Phosphorylation of retinoblastoma protein (pRb) at Thr^821^ is induced by active cyclin A1/Cdk2 complexes and is thereby a measure of Cdk2 activity^35^. Following 16 h VEGF treatment, pRb was increased by 80% in migrating control siRNA-transfected HUVEC, indicative of active Cdk2. Phosphorylation was abrogated by HO-1 silencing, revealing reduced Cdk2 activity and suggesting a link between cell cycle regulation and HO-1-driven endothelial migration (Fig. 5C). To explore these observations *in vivo*, Matrigel, supplemented with VEGF and heparin and containing control siRNA or previously validated siRNAs against murine HO-1 or cyclin A1 (Supplementary Figure 3D,E), was injected sub-cutaneously in female C57Bl/6 mice. After 7 days VEGF supplementation had significantly increased the vascularisation of Matrigel plugs containing control siRNA (from 2.2 ± 0.4 to 17.25 ± 1.57 neovessels per high power field (hpf)), while depletion of HO-1 or cyclin A1 attenuated neovessel development in response to VEGF, reducing neovessels to 12.55 ± 1.81 and 9.45 ± 1.50/hpf respectively (Fig. 6A–F and K). Inclusion of Ad-HO-1 in Matrigel plugs was sufficient to enhance angiogenesis by more than 2-fold to 23.42 ± 3.15/hpf when compared to a control virus (8.89 ± 2.22/hpf) (p < 0.01). Furthermore, the presence of Ad-HO-1 overcame the inhibitory effect of A1 depletion (20.67 ± 2.18/hpf) (Fig. 6G–K), suggesting the existence of additional HO-1-regulated genes involved in angiogenesis.

### HO-1 depletion alters VEGF-induced calpain activity and vimentin cleavage

To identify further HO-1 targets capable of affecting endothelial cell function, the proteome of HUVEC transfected with HO-1 or control siRNA and exposed to VEGF was compared. Cy3 and Cy5-labelled proteins were separated by difference in-gel electrophoresis (Supplementary Figure 4). Subsequent image analysis revealed 29 proteins with a significant difference (p < 0.05, fold change >1.2 or <−1.2) in signal intensity between control and HO-1-depleted cells (Supplementary Table 2 shows all identified proteins and ratio/p-value). These altered proteins were analysed using DAVID and grouped according to gene ontology, focusing on cytoskeletal proteins, intermediate filaments and proteins regulating cell structure and motility (Supplementary Table 3). A common hit amongst all these groups was the intermediate filament vimentin, which plays a role in cell adhesion and migration and has been implicated in the maintenance of angiogenic sprouting and vascular endothelial integrity^36^,^37^. Vimentin was therefore selected for further study.

**Figure 7:**
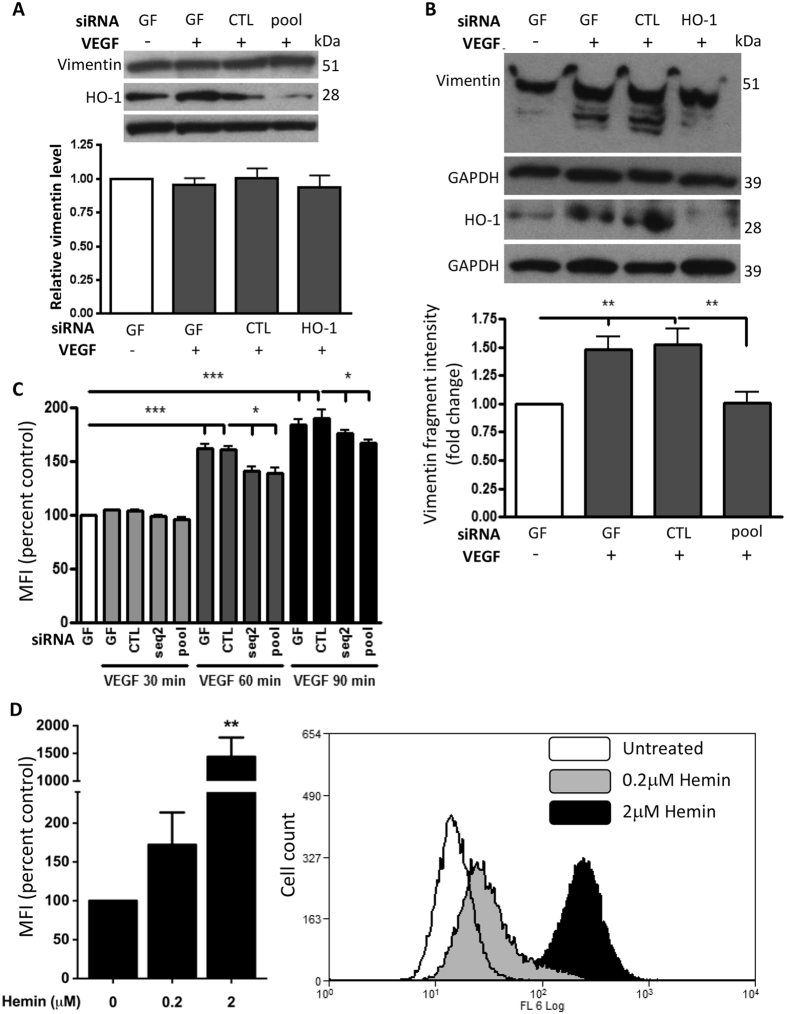
Calpain activation by VEGF is inhibited in HO-1-depleted HUVEC. (**A,B**) HUVEC were treated with geneFECTOR (GF) alone or transfected with control siRNA (CTL) or pooled HO-1 siRNAs (HO-1) prior to treatment with VEGF (25 ng/ml) or vehicle for 16 h and: (**A**) HO-1 and vimentin were quantified by immunoblotting, and in (**B**) Vimentin and HO-1 were assessed by Phos-tag SDS-PAGE and immunoblotting. Changes in expression were quantified by densitometry corrected for GAPDH. (**C**) HUVEC were either treated with GF alone or transfected with CTL or HO-1 siRNAs (seq2 or pool) for 24 h. Cells were pre-loaded with calpain substrate tBoc-LM-CMAC prior to treatment with VEGF or vehicle. In (**D**) HUVEC were pre-loaded with the calpain substrate prior to treatment with hemin (0.2 or 2 μM) for 2 h. Calpain activity was assessed by flow-cytometry. Data are presented as the mean fluorescence intensity (MFI) ± SEM (n = 3 experiments)*p < 0.05,**p < 0.01, ***p < 0.001.

Initial immunoblotting demonstrated no detectable difference in total vimentin between control and HO-1-depleted EC (Fig. 7A), suggesting that the changes in vimentin intensity seen on the 2D gels may reflect its size or phosphorylation status. VEGF induces vimentin cleavage via activation of calpains and further immunoblotting analysis revealed multiple 40–50kDa vimentin bands in VEGF-treated HUVEC (Fig. 7B). This response was not altered by inclusion of a lambda phosphatase (Supplementary Figure 5A), suggesting cleaved vimentin accounted for the 40–50 kDa bands as opposed to differential vimentin phosphorylation. Importantly, HO-1 depletion significantly reduced vimentin cleavage, with fewer lower intensity bands seen (Fig. 7B). Subsequent analysis of HUVEC calpain activity revealed a significant increase after 60–90 mins of VEGF treatment (maximally 60 ± 3.9% at 60 mins, and 93 ± 5.7% above control at 90 mins). This response was partially but significantly attenuated by HO-1 depletion to 39 ± 4.3% at 60 mins and 70 ± 3.7% above control at 90 mins (p < 0.05) (Fig. 7C). Next, to determine whether activation of HO-1 itself can induce calpain activity, HUVEC were treated with a potent HO-1 agonist hemin for 2 hours. As shown in Fig. 7D, hemin led to a concentration-dependent increase in calpain activity, so reinforcing the link between HO-1 and calpain activity.

### HO-1 depletion affects vimentin distribution and endothelial adhesion

**Figure 8:**
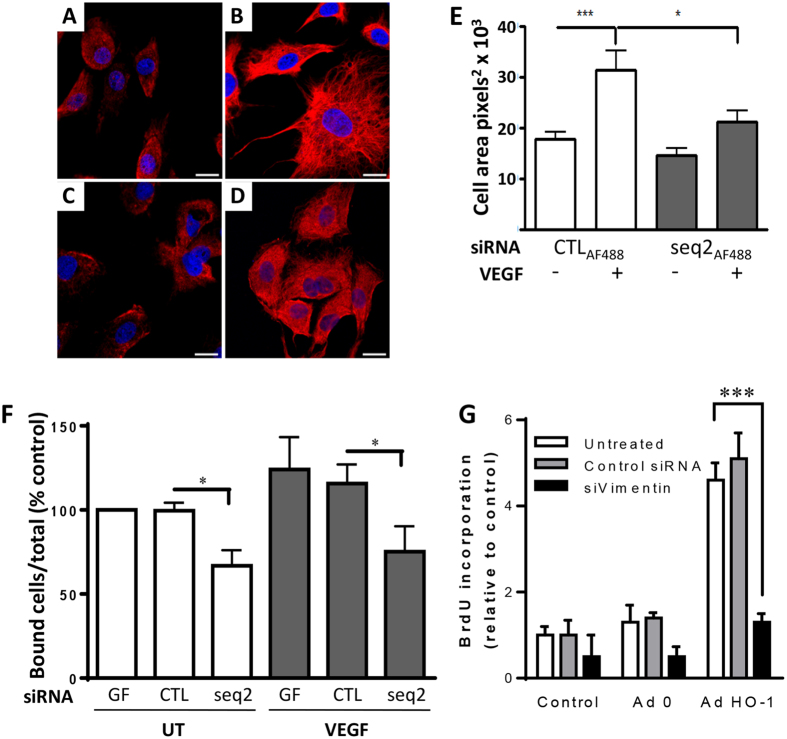
Vimentin filament assembly and EC adhesion is impaired by HO-1-depletion. HUVEC, transfected with (**A,B**) CTLAF_488_ or (**C,D**) HO-1 siRNA seq2AF_488_, were seeded onto gelatin-coated coverslips in the absence (UT) or the presence of 25 ng/ml VEGF for 16 h, prior to fixation and vimentin staining. Nuclei were stained with Draq5. Immunofluorescence was analyzed by confocal microscopy. Representative images are shown, 63x magnification, bar = 50 μm. (**E**) Cell area was quantified using ImageJ (n = 15). (**F**). HUVEC treated with geneFECTOR (GF) alone or transfected with control (CTL) or HO-1 siRNA (seq2) were labelled with 5-chloromethylfluorescein diacetate (6.25 μM) and seeded onto gelatin-coated 96-well plates in the absence (UT) or the presence of VEGF for 40 min. Fluorescence was measured pre- and post-washing, with adhesion expressed as the percentage of bound cells relative to the total number seeded. (**G**). HUVEC were left untreated or transfected with control or vimentin siRNAs. Cells were then left untreated (Control) or infected with Ad0 and AdHO-1 for 24 hrs. Cell proliferation was quantified by BrdU ELISA. Data are presented as mean ± SEM (n = 3–5 experiments), *p < 0.05, ***p < 0.001.

Next, vimentin filament assembly was studied to investigate the implications of HO-1 depletion on vimentin function. Confocal z-stack analysis demonstrated a punctate vimentin staining pattern in control siRNA-transfected EC, consistent with vimentin particles and short filaments. Exposure of these cells to VEGF resulted in the development of a widespread dense vimentin filament network (Fig. 8A,B). Whilst there was no alteration in the vimentin staining pattern or cell area in untreated cells following HO-1-depletion, changes in response to VEGF were significantly attenuated (Fig. 8C–E). VEGF increased cell area from 17.7 ± 1.5 × 10^3^ to 31.3 ± 3.7 × 10^3^ pixels^2^ (p < 0.01), with the latter area reduced to 21.1 ± 2.1 × 10^3^ pixels^2^ in HO-1-depleted cells exposed to VEGF (p < 0.05).

Vimentin regulates EC integrin function and cell adhesion, key components of cell migration^38^. To investigate the potential role of HO-1 and vimentin in EC adhesion, HUVEC loaded with fluorescent dye 5-chloromethylfluorescein diacetate were seeded on to either gelatin or collagen type-1 coated plastic ± VEGF. No effect of HO-1-depletion on β1 integrin-dependent adhesion to collagen was seen (not shown). In contrast, despite no difference in the surface expression of α_v_β_3_ (Supplementary Figure 5B), there was a reduction in adhesion to gelatin of 34% and 41% in the absence and presence of VEGF respectively (p < 0.05), suggesting impaired integrin activation and α_v_β_3_-dependent adhesion (Fig. 8F). Together these data indicate a close relationship between HO-1, calpain activity and vimentin function, with loss of HO-1 resulting in impaired cytoskeletal structure, EC adhesion and migration. Finally, in light of the findings shown in Fig. 6 suggesting the existence of additional HO-1-regulated genes involved in angiogenesis, we investigated the role of vimentin. HUVEC were depleted of vimentin using siRNA prior to adenoviral over-expression of HO-1. While cell proliferation induced by HO-1 was unaffected by control siRNA, silencing of vimentin abrogated the HO-1-mediated proliferative response (p < 0.001).

## Discussion

**Figure 9:**
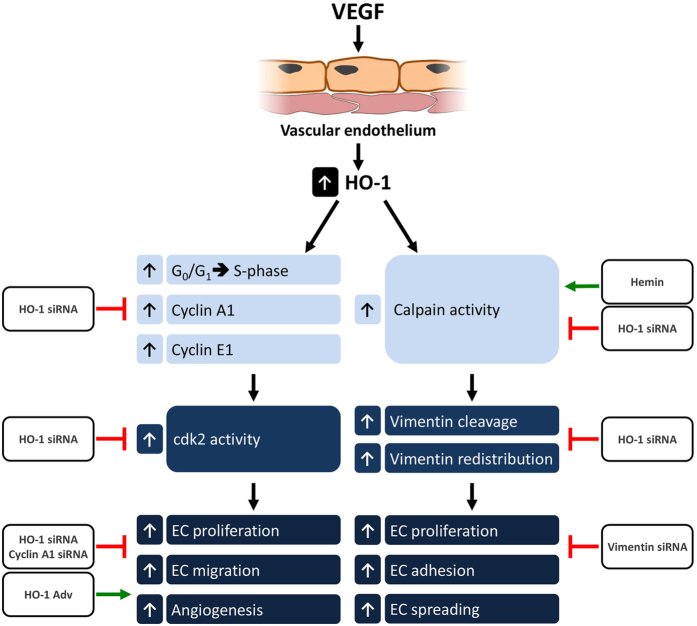
Cyclin A1, E1 and vimentin are downstream targets of HO-1. VEGF treatment of endothelial cells activates HO-1-dependent migration, proliferation and angiogenesis *in vitro* and *in vivo*. Microarray and proteomic analyses revealed cyclin A1, cyclin E1 and vimentin as downstream targets of HO-1. VEGF induced an HO-1-dependent increase in cdk2 kinase and calpain activity. These changes increased cell cycle progression and vimentin cleavage respectively. The role of HO-1 was established using specific siRNAs and HO-1 agonists. Similarly the importance of both cyclin A1 and vimentin in pro-angiogenic effects of VEGF and HO-1 activity were demonstrated by cyclin A1 and vimentin gene silencing.

HO-1 plays a central role in angiogenesis driven by VEGF and SDF1-α. Both cytokines increase HO-1 expression and activity^5^,^16^,^20^,^22^, with the induction of HO-1 dependent upon nuclear translocation of Nrf2^24^. However, a key challenge remains, namely identification of HO-1 target genes, and this was the predominant aim of the current study. Using gain and loss of function approaches we have identified cyclin A1, cyclin E1 and vimentin as downstream targets of HO-1 during VEGF-driven angiogenic responses in the vascular endothelium (Fig. 9). HO-1 depletion inhibited human EC proliferation, tubule formation and directional migration along a VEGF chemotactic gradient; a phenotype reversed by HO-1 over-expression. Silencing of HO-1 in VEGF-treated cells resulted in endothelial cell cycle arrest associated with depletion of cyclins A1 and E1, increased p27 expression and reduced cdk 2 activity. In Matrigel plug assays, HO-1 or cyclin A1 siRNA inhibited VEGF-driven angiogenesis. Proteomic analysis identified vimentin as a downstream target of HO-1 in VEGF-treated human EC. VEGF-induced calpain activity and subsequent cleavage of vimentin was abrogated by HO-1 siRNA. VEGF-mediated changes in vimentin filament assembly and αvβ3-dependent adhesion of EC were also attenuated, while vimentin-depletion abrogated HO-1 driven EC proliferation.

In previous studies, retroviral over-expression of HO-1 in EC enhanced expression of cyclins A1 and E1, increasing proliferation and the percentage of cells in the G1 phase of the cell cycle^39^. The cyclin-dependent kinase inhibitors p21 and p27 were decreased by over-expression^14^,^39^. We have now shown that induction of cyclins A1 and E1 by VEGF requires HO-1, and that HO-1 depletion results in cell cycle arrest in human EC. Of note, cyclin A1 has also been linked with VEGF-synthesis in prostate cancer^40^. Thus, we propose that positive feedback and endogenous amplification between VEGF and HO-1 in EC^23^ is driven by a VEGF-HO-1-cyclin A1-VEGF feedback loop. During inflammation this mechanism is important for vascular homeostasis, angiogenesis and tissue repair. However, in another context, such as prostate or lung carcinomas, the feedback loop may contribute to the link between HO-1 activity, carcinogenesis, tumour growth and metastasis^41^,^42^,^43^.

Cyclin A1 has also been implicated in EC migration, a critical step in angiogenesis. Contact-inhibited EC exhibit low-levels of cyclin A1 mRNA and protein^44^,^45^, with reduced cyclin A1, cyclin E1 and cdk2 kinase activity reported^44^. Following scratch injury, exposure of EC to VEGF led to cyclin A1 induction and an increase in cdk2 activity, as indicated by enhanced phosphorylation of RB protein (Fig. 5). Thus, reduced cdk2 activity represents a likely mechanism for the inhibition of VEGF-mediated EC proliferation and migration seen in HO-1-depleted HUVEC. HO-1 silencing coincided with a fall in cyclin A1 and E1 and an increase in the inhibitory regulator p27, moving the cells back towards the quiescent phenotype observed following contact-inhibition. *In vivo* analyses revealed a role for HO-1^5^ and cyclin A1 in VEGF-driven neovessel development. The inhibitory effect of cyclin A1 siRNA was reversed by HO-1 over-expression. This outcome may reflect incomplete depletion of cyclin A1 by siRNA in this setting. Alternatively, the presence of AdHO-1 may upregulate cyclin E1 or other pro-proliferative target genes, increase VEGF synthesis by EC or infiltrating monocytes, and/or drive additional pathways such as those regulated by SDF-1α.

Proteomic analysis sought additional HO-1 targets important for VEGF-driven angiogenesis, with vimentin emerging as a potential candidate. Vimentin is a highly conserved intermediate filament family protein, abundant in EC and mesenchymal cells. Dynamic interactions between vimentin, and actin-containing microfilaments and tubules stabilises cell structure. Beyond this, the study of vimentin-deficient mice has revealed novel functions in the vascular endothelium including maintenance of monolayer integrity, control of leukocyte trafficking and angiogenesis^37^,^46^,^47^. A central component is the role of vimentin in cell migration. Vimentin regulates cell-cell contact and adhesion through an association with hemidesmosomes, and via control of integrin function. In EC, vimentin interacts with integrin α_v_β_3_ in vimentin-associated matrix adhesions (VAMs)^48^. VAMs are actively assembled at the forefront of migrating cells and are disrupted by siRNA depletion of vimentin which leads to loss of cell adhesion^49^. In fibroblasts and vascular smooth muscle cells vimentin plays a central role in cell motility^50^,^51^.

Compared to control transfected cells, HO-1-depleted EC exhibited reduced adhesion to gelatin and impaired migration. The VEGF-induced switch from non-filamentous particles/short vimentin filaments to long, mature and peripheral filaments was attenuated by HO-1 silencing. Although rapid phosphorylation-dephosphorylation events play an important role in the regulation of vimentin filament distribution, no change in vimentin phosphorylation following HO-1 depletion was detected. To explore this further, we turned to the recent report that growth factor-induced calpain activation results in vimentin cleavage and relocalisation to the EC periphery, a response required for angiogenic sprouting^36^. Calpains are calcium-dependent cysteine proteases. They control cell migration through cleavage of actin regulatory proteins and participate in cell cycle regulation^52^,^53^. HO-1 depletion attenuated VEGF-induced calpain activity and the associated cleavage and redistribution of vimentin filaments. Furthermore, pharmacological activation of HO-1 by hemin proved to be a potent inducer of calpain activity. We propose that loss of this close association between calpain, vimentin and HO-1 is a significant factor in the inhibition of cell migration observed in HO-1 deficient EC.

Importantly silencing of vimentin attenuated the pro-proliferative effect of HO-1 over-expression in HUVEC. The suggestion of a relationship between HO-1 activity and vimentin is supported by phenotypic similarities between the knockout mice. *Hmox*1^−/−^ mice exhibit endothelial dysfunction, defective endothelial progenitor homing, reduced neovascularisation during wound healing and attenuated re-endothelialisation of the retinal vasculature following ischaemic injury^16^,^17^,^54^. Likewise, vimentin-deficient animals suffer impaired hypoxia-induced retinal neovascularisation, defects in endothelial barrier function and poor wound healing, which are associated with dysregulated granulation tissue generation, a process requiring angiogenic sprouting^37^,^46^,^47^.

In conclusion, the role of HO-1 during angiogenesis is context-dependent, important and offers therapeutic opportunities. However, a detailed understanding of the specific pro- and anti-angiogenic effects of HO-1, its enzymatic products and downstream target genes in regulating the the angiogenic cascade, for example during wound healing or in pathologies such as chronic inflammatory disease or neoplasms, is essential if therapeutic targeting is ever to be achieved safely^2^,^55^.

## Additional Information

**How to cite this article**: Bauer, A. *et al*. Identification of cyclins A1, E1 and vimentin as downstream targets of heme oxygenase-1 in vascular endothelial growth factor-mediated angiogenesis. *Sci. Rep.* **6**, 29417; doi: 10.1038/srep29417 (2016).

## Supplementary Material

Supplementary Information
