# Supplementary material for: Identification of cyclins A1, E1 and vimentin as downstream targets of heme oxygenase-1 in vascular endothelial growth factor-mediated angiogenesis
Source: Sci Rep. 2016 Jul 8;6:29417. doi: 10.1038/srep29417 (PMC4937390; doi:10.1038/srep29417)
Supplement: Supplementary Information [file srep29417-s1.pdf]

Identification of cyclins A1, E1 and vimentin as downstream targets of heme oxygenase-1 in vascular endothelial growth factor-mediated angiogenesis

Andrea Bauer[1], Hayley Mylroie[1*], C Clare Thornton[1*], Damien Calay[1*], Graeme M Birdsey[1],

Allan P Kiprianos[1], Garrick K Wilson[1], Miguel P Soares[2], Xiaoke Yin[3], Manuel Mayr[3],

Anna M Randi[1], Justin C Mason[1].

Supplementary Material

Supplementary Figure Legends

Supplementary Figure 1. Effect of HO-1 depletion on VEGF-induced EC proliferation and cytoprotection. HUVEC were transfected with geneFECTOR (GF) alone, control siRNA (CTL), or HO-1 siRNAs (seq2, pool) prior to treatment with hemin (0.2 μM) or vehicle for 6h. (A) immunoblotting of HO-1 and tubulin with densitometric quantification of HO-1 relative to hemin-treated positive control EC. (C) HUVEC were transfected with $CTLAF_{488}$ or HO-1 siRNA $seq2AF_{488}$ prior to immunoblotting for HO-1, HO-2 and tubulin. (C) HUVEC were left uninfected or infected with Ad-HO-1 or Ad0 for 24 h prior to immunoblotting of HO-1 and tubulin. (D and E) HUVEC were left untreated or treated with VEGF (25 ng/ml), ZnPP (5-20 μM), or VEGF and ZnPP in combination. (D) EC proliferation was quantified by BrdU incorporation after 48h, and (E) cell number by MTT assay after 24 h. Data are presented as mean ± SEM (n=3 experiments). *$p<0.05$, **$p<0.01$, ***$p<0.001$ versus UT control. δδ $p<0.01$ and ns = non-significant versus VEGF-treated positive control.

Supplementary Figure 2. HO-1 inhibition impairs microtubule formation. Human microvascular EC (HMEC-1) were transfected with $CTLAF_{488}$ or HO-1 siRNAs ($seq2AF_{488}$). (A) confluent EC monolayers were scratched and migration distance in the absence or presence of VEGF (25 ng/ml) for 16h was assessed by live cell imaging. (B) HMEC-1 were transfected with control or HO-1 siRNA (seq2, pool) for 24h prior to harvesting and re-seeding onto Matrigel in the absence or presence of VEGF (25 ng/ml) for 6 h and visualised using phase-contrast microscopy. Images from five fields of view were captured and tube length quantified using ImageJ (NeuronJ). Data are presented as mean ± SEM (n=3 experiments), **$p<0.01$, ***$p<0.001$.

Supplementary Figure 3. HO-1 inhibition senescence and cyclin regulation. (A) HUVEC transfected with CTL or HO-1 siRNAs (seq2 or pool) were left untreated or treated with VEGF (25 ng/ml) for 48 h. Senescent cells were identified by SA-β-gal staining. HUVEC cultured for 12 passages (P12) acted as a positive control. (B-C) HUVEC were treated with geneFECTOR (GF) alone or transfected with CTL or HO-1 siRNAs prior to treatment with VEGF (25 ng/ml) or vehicle. (B) cyclin B1 and (C) cyclin D1 mRNAs were quantified by qRT-PCR. (D-E) Murine EC were treated with GF alone or transfected with CTL, cyclin A1 or HO-1 siRNAs and after 48 h (D) HO-1 and (E) cyclin A1 mRNAs were quantified by qRT-PCR. Data are presented as mean ± SEM (n=3-5 experiments). ns = not significant and $^{\delta}p<0.05$ versus untreated CTL-transfected cells.

Supplementary Figure 4. **Difference in gel electrophoresis.** A representative DIGE fluorescent image showing the overlay of Cy5-labelled Ctrl siRNA + VEGF (red) and Cy3-labelled HO-1 siRNA + VEGF (green).

Supplementary Figure 5. Vimentin band phosphorylation and $\alpha_V\beta_3$ expression. (A) HUVEC were treated with geneFECTOR (GF) alone or transfected with CTL or HO-1 siRNAs prior to treatment with VEGF (25 ng/ml) or vehicle for 16h. Protein lysates were treated with lambda phosphatase (λ PP, 200 units). Vimentin and GAPDH were analysed by by Phos-tag^{TM} SDS-PAGE and immunoblotting. A representative blot from two separate experiments is shown. (B) HUVEC were transfected as above prior to treatment with VEGF or vehicle for 40 minutes. Integrin $\alpha_V\beta_3$ expression was analysed by flow-cytometry and presented as relative fluorescent intensity, normalized to EC treated with GF alone. Data are shown as mean ± SEM (n=4 experiments), ns=not significant.

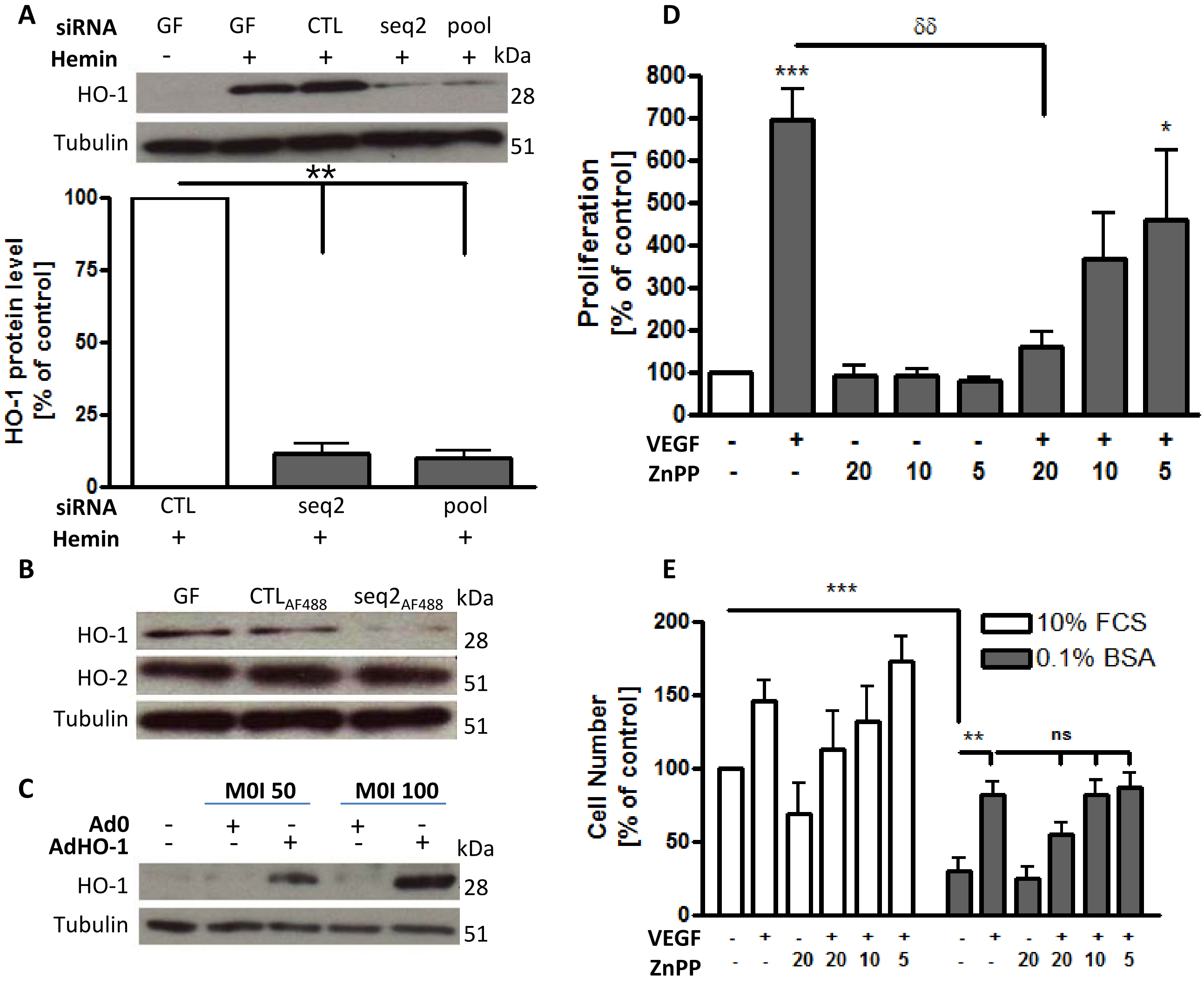

Supplementary Figure 1

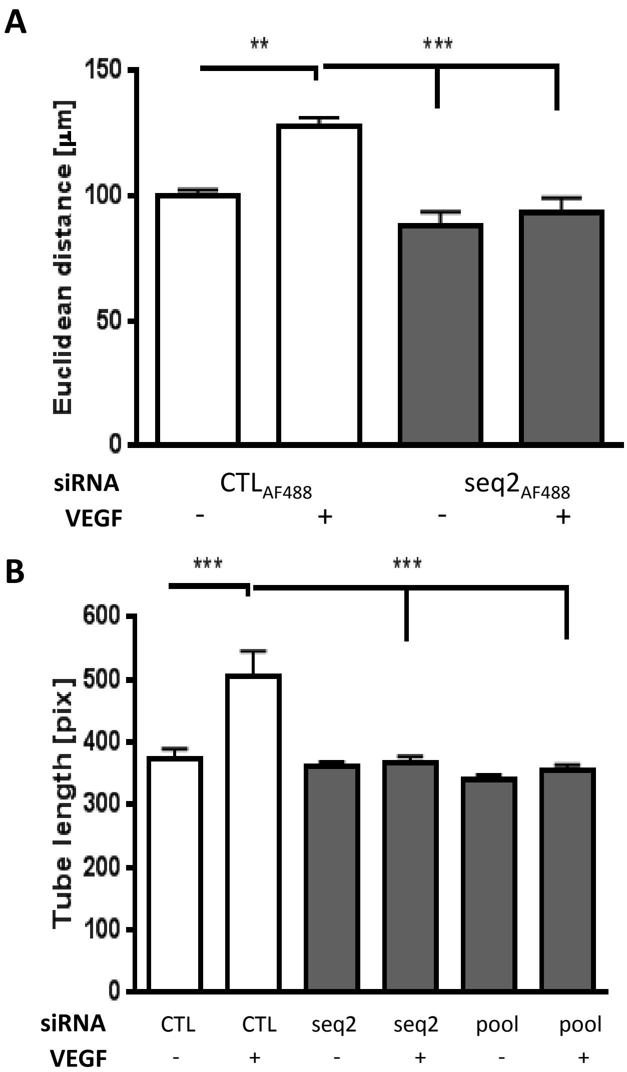

Supplementary Figure 2

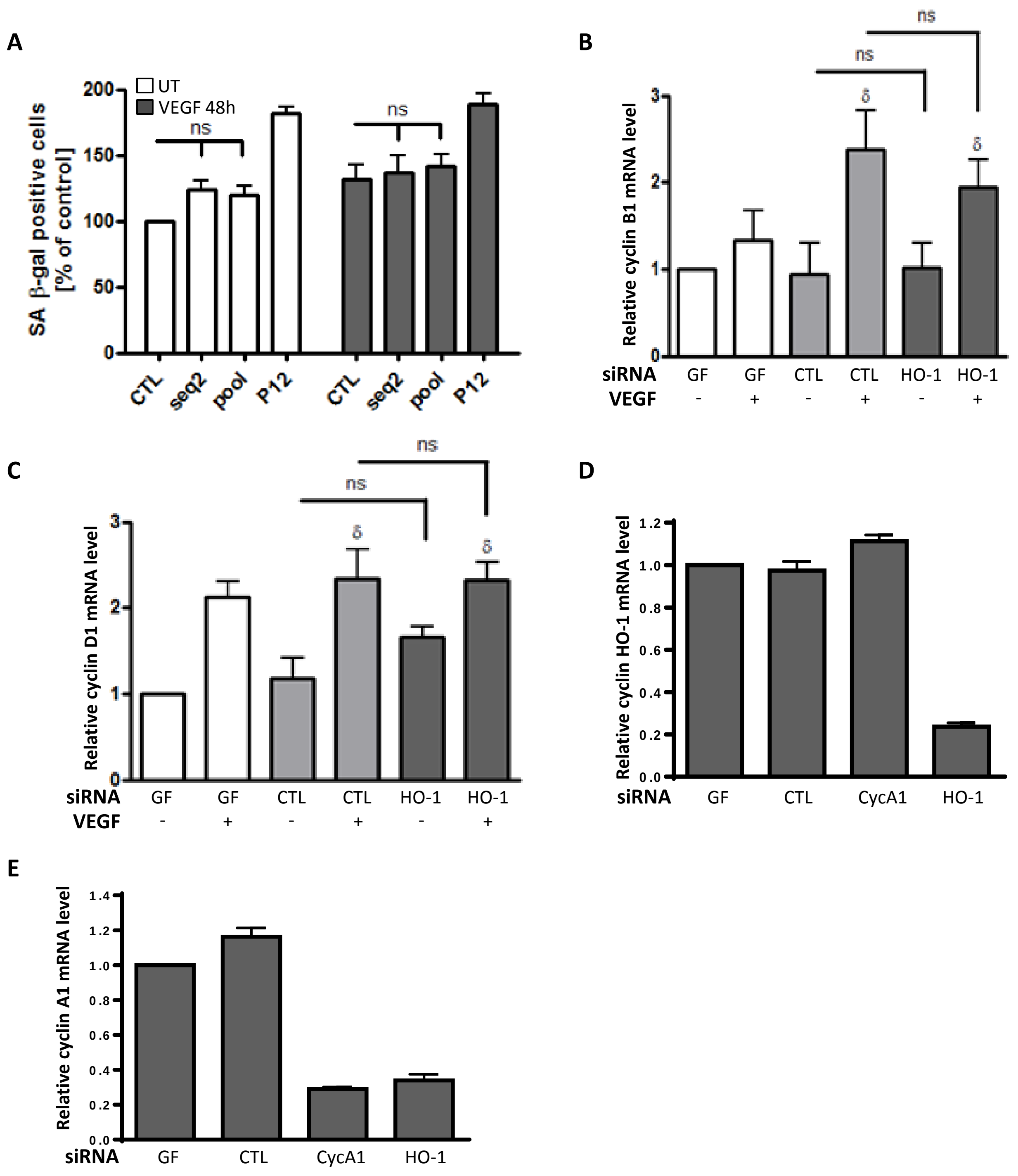

Supplementary Figure 3

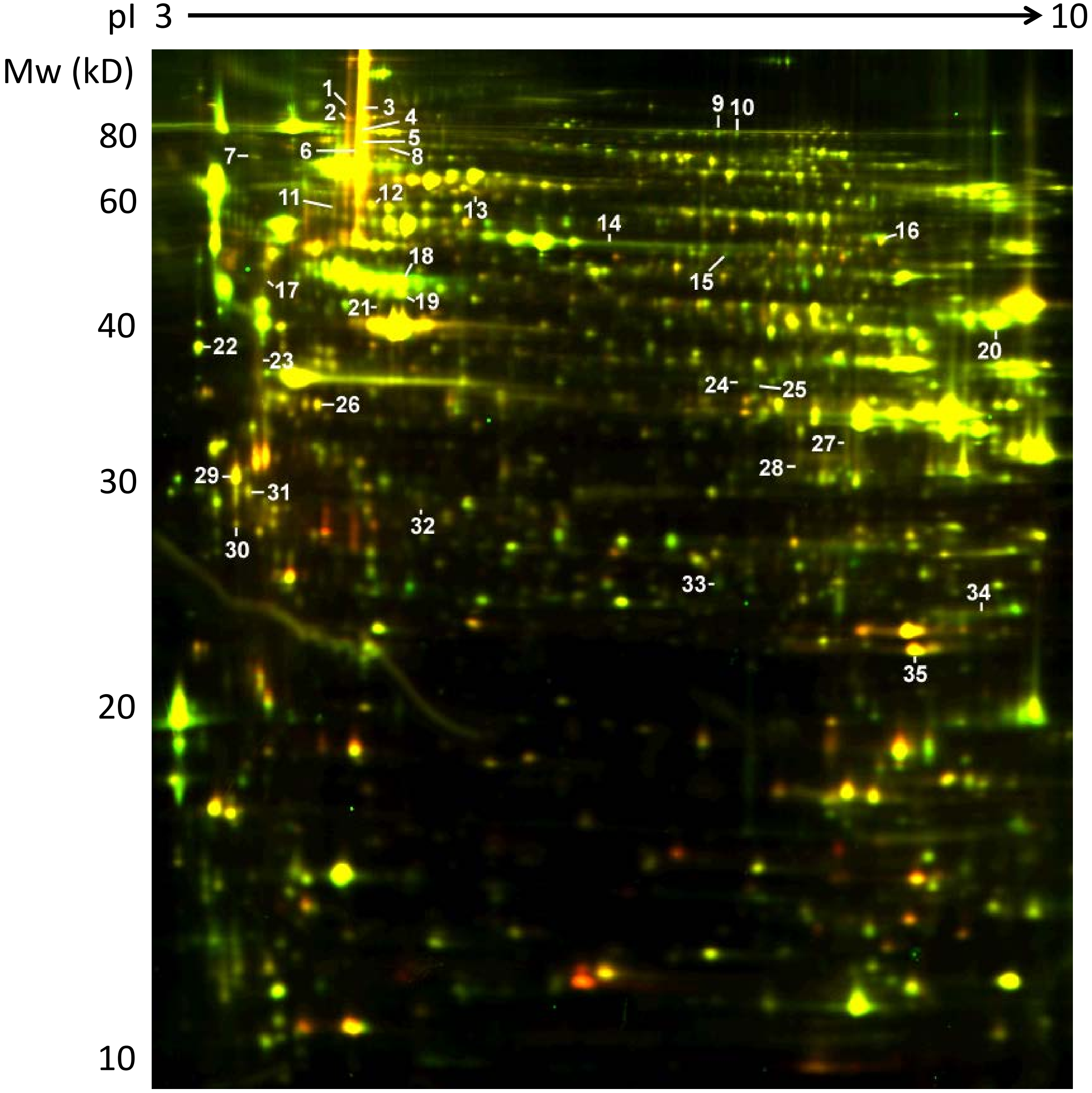

Supplementary Figure 4

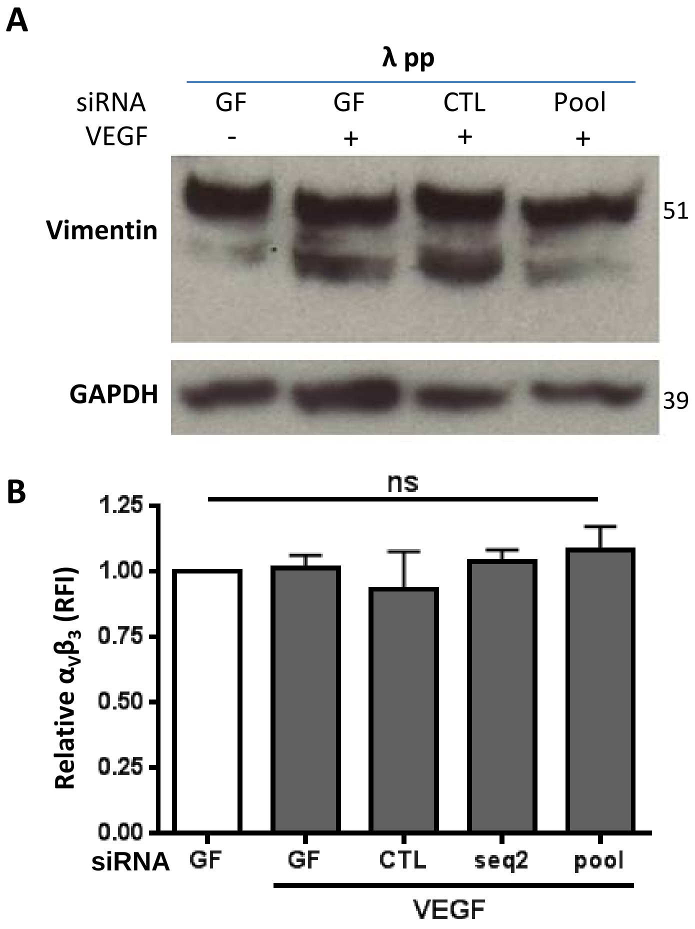

Supplementary Figure 5

Supplementary Table 1: Cytoskeleton Regulator PCR array results. Changes in mRNA expression of siCTL- or siHO-1 siRNA-transfected HUVEC in response to VEGF were assessed using a PCR Array. Upregulation of gene expression ≥ 2.00 fold and downregulation ≤ 0.50 fold were considered significant changes in gene expression.

Gene Symbol	Gene Name	Fold Up- or Down-Regulation
ACTR2	ARP2 actin-related protein 2 homolog (yeast)	1.01
ACTR3	ARP3 actin-related protein 3 homolog(yeast)	0.77
ARFIP2	ADP-ribosylation factor interacting protein 2	1.34
ARHGAP6	Rho GTPase activating protein 6	1.16
ARHGDIB	Rho GDP dissociation inhibitor (GDI) beta	0.82
ARHGEF11	Rho guanine nucleotide exchange factor (GEF) 11	1.43
ARPC1B	Actin related protein 2/3 complex, subunit 1B, 41kDa	1.34
**ARPC2**	**Actin related protein 2/3 complex, subunit 2, 34kDa**	**2.17**
ARPC3	Actin related protein 2/3 complex, subunit 3, 21kDa	0.82
ARPC4	Actin related protein 2/3 complex, subunit 4, 20kDa	1.54
ARPC5	Actin related protein 2/3 complex, subunit 5, 16kDa	0.58
AURKA	Aurora kinase A	1.43
AURKB	Aurora kinase B	1.34
AURKC	Aurora kinase C	1.65
BAIAP2	BAI1-associated protein 2	1.09
CALD1	Caldesmon 1	0.88
CALM1	Calmodulin 1 (phosphorylase kinase, delta)	0.95
CASK	Calcium/calmodulin-dependent serine protein kinase (MAGUK family)	1.09
**CCNA1**	**Cyclin A1**	**0.44**
CCNB2	Cyclin B2	1.43
CDC42	Cell division cycle 42 (GTP binding protein, 25kDa)	0.95
CDC42BPA	CDC42 binding protein kinase alpha (DMPK-like)	1.25
CDC42EP2	CDC42 effector protein (Rho GTPase binding) 2	1.01
CDC42EP3	CDC42 effector protein (Rho GTPase binding) 3	1.16
CDK5	Cyclin-dependent kinase 5	1.43
CDK5R1	Cyclin-dependent kinase 5, regulatory subunit 1 (p35)	1.43
ARAP1	ArfGAP with RhoGAP domain, ankyrin repeat and PH domain 1	1.25
CFL1	Cofilin 1 (non-muscle)	1.01
**CIT**	**Citron (rho-interacting, serine/threonine kinase 21)**	**2.03**
CLASP1	Cytoplasmic linker associated protein 1	0.88
CLASP2	Cytoplasmic linker associated protein 2	0.88
CLIP1	CAP-GLY domain containing linker protein 1	1.09
CLIP2	CAP-GLY domain containing linker protein 2	1.25
CRK	V-crk sarcoma virus CT10 oncogene homolog (avian)	0.82
CTTN	Cortactin	1.01
CYFIP1	Cytoplasmic FMR1 interacting protein 1	0.95
CYFIP2	Cytoplasmic FMR1 interacting protein 2	1.77
DIAPH1	Diaphanous homolog 1 (Drosophila)	0.95
DSTN	Destrin (actin depolymerizing factor)	1.01
EZR	Ezrin	1.65
FNBP1L	Formin binding protein 1-like	1.16
**FSCN2**	**Fascin homolog 2, actin-bundling protein, retinal (Strongylocentrotus purpuratus)**	**2.17**

Supplementary Figure 4 (continued)

GSN	Gelsolin (amyloidosis, Finnish type)	1.09
IQGAP1	IQ motif containing GTPase activating protein 1	0.88

IQGAP2	IQ motif containing GTPase activating protein 2	1.09
LIMK1	LIM domain kinase 1	1.09
LIMK2	LIM domain kinase 2	0.82
LLGL1	Lethal giant larvae homolog 1 (Drosophila)	1.25
MACF1	Microtubule-actin crosslinking factor 1	0.82
MAP3K11	Mitogen-activated protein kinase kinase kinase 11	1.54
MAP4	Microtubule-associated protein 4	0.72
MAPK13	Mitogen-activated protein kinase 13	1.16
MAPRE1	Microtubule-associated protein, RP/EB family, member 1	0.82
MAPRE2	Microtubule-associated protein, RP/EB family, member 2	0.58
MAPT	Microtubule-associated protein tau	0.72
MARK2	MAP/microtubule affinity-regulating kinase 2	1.09
MID1	Midline 1 (Opitz/BBB syndrome)	0.88
MSN	Moesin	0.62
MYLK	Myosin light chain kinase	1.16
MYLK2	Myosin light chain kinase 2	1.34
NCK1	NCK adaptor protein 1	1.01
NCK2	NCK adaptor protein 2	1.01
PAK1	P21 protein (Cdc42/Rac)-activated kinase 1	1.43
PAK4	P21 protein (Cdc42/Rac)-activated kinase 4	1.09
PFN2	Profilin 2	1.09
PHLDB2	Pleckstrin homology-like domain, family B, member 2	0.95
PIKFYVE	Phosphoinositide kinase, FYVE finger containing	1.16
**PPP1R12A**	Protein phosphatase 1, regulatory (inhibitor) subunit 12A	1.43
PPP1R12B	**Protein phosphatase 1, regulatory (inhibitor) subunit 12B**	**2.03**
PPP3CA	Protein phosphatase 3 (formerly 2B), catalytic subunit, alpha isoform	0.82
PPP3CB	Protein phosphatase 3 (formerly 2B), catalytic subunit, beta isoform	0.51
RAC1	Ras-related C3 botulinum toxin substrate 1 (rho family, small GTP binding protein Rac1)	0.88
RACGAP1	Rac GTPase activating protein 1	1.25
RDX	Radixin	0.82
RHOA	Ras homolog gene family, member A	0.82
ROCK1	Rho-associated, coiled-coil containing protein kinase 1	0.95
SSH1	Slingshot homolog 1 (Drosophila)	1.16
SSH2	Slingshot homolog 2 (Drosophila)	0.77
STMN1	Stathmin 1	1.09
TIAM1	T-cell lymphoma invasion and metastasis 1	1.65
VASP	Vasodilator-stimulated phosphoprotein	1.01
WAS	Wiskott-Aldrich syndrome (eczema-thrombocytopenia)	0.51
WASF1	WAS protein family, member 1	1.01
WASL	Wiskott-Aldrich syndrome-like	1.16

Supplementary Table 2. Significantly altered protein spots identified by DIGE LC-MS/MS.

No.	Protein name	Accession No.	Mw (Da)	p value	Average Ratio*	# unique peptides	# unique spectra	# total spectra	Sequence coverage
**1**	Vimentin	VIME_HUMAN	53,635	0.013	-2.03	16	18	30	36.30%
**2**	Vimentin	VIME_HUMAN	53,635	0.016	-1.71	21	25	43	48.30%
**3**	Vimentin	VIME_HUMAN	53,635	0.0024	-1.69	35	48	90	68.70%
**4**	Vimentin	VIME_HUMAN	53,635	0.0067	-1.65	38	44	81	79.20%
**5**	Vimentin	VIME_HUMAN	53,635	0.0068	-1.63	34	40	69	71.00%
**6**	Vimentin	VIME_HUMAN	53,635	0.0047	-1.66	34	40	76	72.50%
**7**	Periodic tryptophan protein 1 homolog	PWP1_HUMAN	55,811	0.017	-1.2	3	3	5	6.39%
**8**	Lamin-B1	LMNB1_HUMAN	66,392	0.0054	-1.22	4	4	5	7.85%
**8**	Ran GTPase-activating protein 1	RAGP1_HUMAN	63,525	0.0054	-1.22	4	4	6	7.84%
**9**	Procollagen-lysine,2-oxoglutarate 5-dioxygenase 2	PLOD2_HUMAN	84,669	0.0046	1.2	8	9	17	13.00%
**10**	Nucleolin	NUCL_HUMAN	76,598	0.00056	1.21	3	3	6	4.37%
**10**	Procollagen-lysine,2-oxoglutarate 5-dioxygenase 2	PLOD2_HUMAN	84,669	0.00056	1.21	9	9	14	13.70%
**11**	Vimentin	VIME_HUMAN	53,635	0.0018	-1.91	10	10	16	24.00%
**12**	Heterogeneous nuclear ribonucleoprotein K	HNRPK_HUMAN	50,961	0.028	-1.24	7	11	19	17.90%
**13**	Ras GTPase-activating protein-binding protein 1	G3BP1_HUMAN	52,145	0.02	-1.26	3	3	5	6.22%
**14**	Protein disulfide-isomerase A3	PDIA3_HUMAN	56,767	0.026	1.25	11	14	23	23.40%
**15**	Succinyl-CoA:3-ketoacid-coenzyme A transferase 1, mitochondrial	SCOT1_HUMAN	56,141	0.01	1.38	5	6	9	10.40%
**16**	Pyruvate kinase isozymes M1/M2	KPYM_HUMAN	57,920	0.043	1.23	10	10	19	17.50%
**17**	Vimentin	VIME_HUMAN	53,635	0.03	-1.52	9	9	15	15.50%
**18**	Thioredoxin domain-containing protein 5	TXND5_HUMAN	47,611	0.044	1.36	16	18	31	35.20%
**19**	Heterogeneous nuclear ribonucleoprotein F	HNRPF_HUMAN	45,654	0.012	1.21	8	11	20	23.40%
**20**	Serpin H1	SERPH_HUMAN	46,424	0.012	1.37	17	22	44	40.90%
**21**	Eukaryotic translation initiation factor 3 subunit F	EIF3F_HUMAN	37,546	0.039	-1.28	6	7	13	21.60%
**21**	Perilipin-3	PLIN3_HUMAN	47,028	0.039	-1.28	5	5	9	18.40%
**22**	Protein SET	SET_HUMAN	33,471	0.037	1.23	7	11	18	22.40%

**23**	Tropomyosin alpha-1 chain	TPM1_HUMAN	32,692	0.043	-1.29	5	5	7	15.50%
**23**	Vimentin	VIME_HUMAN	53,635	0.043	-1.29	4	4	7	7.51%
**24**	Ubiquitin fusion degradation protein 1 homolog	UFD1_HUMAN	34,483	0.047	1.25	3	4	7	7.82%
**25**	Heterogeneous nuclear ribonucleoproteins C1/C2	HNRPC_HUMAN	33,653	0.02	1.36	5	5	7	13.40%
**26**	Elongation factor 1-delta	EF1D_HUMAN	31,104	0.018	-1.24	13	15	25	47.00%
**27**	Voltage-dependent anion-selective channel protein 2	VDAC2_HUMAN	31,549	0.018	-1.24	6	6	10	25.50%
**28**	Voltage-dependent anion-selective channel protein 2	VDAC2_HUMAN	31,549	0.026	-1.31	2	2	3	6.80%
**29**	Elongation factor 1-delta	EF1D_HUMAN	31,104	0.027	-1.23	3	3	4	7.12%
**30**	Elongation factor 1-beta	EF1B_HUMAN	24,746	0.027	-1.23	4	5	7	23.10%
**30**	Eukaryotic translation initiation factor 6	IF6_HUMAN	26,580	0.05	-1.2	6	7	13	29.40%
**31**	14-3-3 protein epsilon	1433E_HUMAN	29,157	0.018	-1.29	12	16	30	35.30%
**32**	Prohibitin	PHB_HUMAN	29,787	9.40E-06	-1.24	7	7	13	22.80%
**33**	LDLR chaperone MESD	MESD_HUMAN	26,060	0.0098	1.41	7	8	16	32.50%
**34**	Cysteine and glycine-rich protein 1	CSRP1_HUMAN	20,549	0.038	1.27	9	10	17	50.80%
**35**	Transgelin-2	TAGL2_HUMAN	22,374	0.047	-1.43	9	13	23	44.20%

* Average Ratio is HO-1 siRNA + VEGF / Control siRNA + VEGF. Positive value indicates increased in HO-1 siRNA + VEGF group. Negative value indicates decreased in HO-1 siRNA + VEGF group.

Supplementary Table 2. Proteins differentially expressed between HO-1 deficient and control human EC. 29 identified proteins were analysed and grouped according to gene ontology (GO) using DAVID Bioinformatics Resources 6.7.

**Cellular Component**<br>**GO Term**	**Count**	**Gene Name**
intracellular organelle lumen	12	SCOT1<br>SET<br>IF6<br>HNRPF<br>HNPRK<br>LMNB1<br>NUCL<br>PHB<br>PDIA3<br>SERPH<br>TXNDC5<br>VDAC2
organelle lumen	12	SCOT1<br>SET<br>IF6<br>HNRPF<br>HNPRK<br>LMNB1<br>NUCL<br>PHB<br>PDIA3<br>SERPH<br>TXNDC5<br>VDAC2
membrane-enclosed lumen	12	SCOT1<br>SET<br>IF6<br>HNRPF<br>HNPRK<br>LMNB1<br>NUCL<br>PHB<br>PDIA3<br>SERPH<br>TXNDC5<br>VDAC2
nuclear lumen	7	SET<br>IF6<br>HNRPF<br>HNPRK<br>LMNB1<br>NUCL<br>PHB
spliceosome	3	HNRPC<br>HNRPF<br>HNPRK
ribonucleoprotein complex	4	HNRPC<br>HNRPF<br>HNPRK<br>NUCL
nucleoplasm	5	SET<br>HNRPF<br>HNPRK<br>NUCL<br>PHB
nucleolus	4	IF6<br>HNRPF<br>HNPRK<br>NUCL
organelle envelope	6	RAGP1<br>IF6<br>LMNB1<br>PHB<br>TAGL2<br>VDAC2

**Supplementary Table 2. (cont)**		
envelope	6	RAGP1<br>IF6<br>LMNB1<br>PHB<br>TAGL2<br>VDAC2
nuclear envelope	4	RAGP1<br>IF6<br>LMNB1<br>TAGL2
nuclear membrane	3	IF6<br>LMNB1<br>TAGL2
organelle inner membrane	3	IF6<br>LMNB1<br>PHB
organelle membrane	5	IF6<br>LMNB1<br>PHB<br>TAGL2<br>VDAC2
endomembrane system	4	RAGP1<br>IF6<br>LMNB1<br>TAGL2
endoplasmic reticulum lumen	3	PDIA3<br>SERPH<br>TXNDC5
endoplasmic reticulum	6	SET<br>MESD<br>PLOD2<br>PDIA3<br>SERPH<br>TXNDC5
endoplasmic reticulum part	3	SET<br>MESD<br>PDIA3<br>SERPH<br>TXNDC5
integral to membrane	3	RAGP1<br>PHB<br>VDAC2
intrinsic to membrane	3	RAGP1<br>PHB<br>VDAC2
intermediate filament	3	IF6<br>LMNB1<br>**VIME**
intermediate filament cytoskeleton	3	IF6<br>LMNB1<br>**VIME**
non-membrane-bound organelle	9	RAGP1<br>HNRPK<br>HNRPF<br>IF6<br>LMNB1<br>NUCL<br>TPM1<br>VDAC2<br>**VIME**
intracellular non-membrane bound organelle	9	RAGP1<br>HNRPK<br>HNRPF<br>IF6<br>LMNB1<br>NUCL<br>TPM1<br>VDAC2<br>**VIME**

**Supplementary Table 2. (cont)**		
cytoskeletal part	4	IF6<br>LMNB1<br>TPM1<br>**VIME**
cytoskeleton	4	IF6<br>LMNB1<br>TPM1<br>**VIME**
mitochondrial part	3	SCOT1<br>PHB<br>VDAC2
mitochondrion	4	SCOT1<br>PHB<br>1433E<br>VDAC2
cytosol	9	G3BP1<br>SET<br>EF1B<br>EF1D<br>EIF3F<br>1433E<br>KPYM<br>UFD1<br>**VIME**
plasma membrane	7	G3BP1<br>RAGP1<br>PLIN3<br>PHB<br>TAGL2<br>TPM1<br>**VIME**
plasma membrane part	4	RAGP1<br>PLIN3<br>PHB<br>TPM1
**Molecular Function**<br>**GO Term**	**Count**	**Gene Name**
translation factor activity, nucleic acid binding		
RNA binding	5	G3BP1<br>HNRPC<br>HNRPK<br>HNRPF<br>NUCL
nucleotide binding	5	G3BP1<br>HNRPC<br>HNRPF<br>NUCL<br>VDAC2
DNA binding	3	G3BP1<br>HNRPK<br>NUCL
structural molecule activity	3	LMNB1<br>TPM1<br>**VIME**
metal ion binding	3	CSRP1<br>PLOD2<br>KPYM
cation binding	3	CSRP1<br>PLOD2<br>KPYM
ion binding	3	CSRP1<br>PLOD2<br>KPYM

Supplementary Table 2. (cont)		
**Biological Process**<br>**GO Term**	**Count**	**Gene Name**
translation	4	EF1B<br>EF1D<br>EIF3F<br>IF6
RNA splicing, via transesterification reactions	3	HNRPC<br>HNRPF<br>HNPRK
nuclear mRNA splicing, via spliceosome	3	HNRPC<br>HNRPF<br>HNPRK
RNA splicing	3	HNRPC<br>HNRPF<br>HNPRK
mRNA processing	3	HNRPC<br>HNRPF<br>HNPRK
mRNA metabolic process	3	HNRPC<br>HNRPF<br>HNPRK
RNA processing	3	HNRPC<br>HNRPF<br>HNPRK
intracellular transport	5	SET<br>PDIA3<br>1433E<br>TXDN5<br>TPM1
regulation of apoptosis	4	PHB<br>PDIA3<br>1433E<br>TXDN5
regulation of programmed cell death	4	PHB<br>PDIA3<br>1433E<br>TXDN5
regulation of cell death	4	PHB<br>PDIA3<br>1433E<br>TXDN5
cell motion	**3**	**1433E**<br>TPM1<br>**VIME**
negative regulation of macromolecule metabolic process	3	SET<br>PHB<br>1433E
